# Overexpression of Peroxiredoxin 4 Affects Intestinal Function in a Dietary Mouse Model of Nonalcoholic Fatty Liver Disease

**DOI:** 10.1371/journal.pone.0152549

**Published:** 2016-04-01

**Authors:** Aya Nawata, Hirotsugu Noguchi, Yuichi Mazaki, Toshihiro Kurahashi, Hiroto Izumi, Ke-Yong Wang, Xin Guo, Hidetaka Uramoto, Kimitoshi Kohno, Hatsumi Taniguchi, Yoshiya Tanaka, Junichi Fujii, Yasuyuki Sasaguri, Akihide Tanimoto, Toshiyuki Nakayama, Sohsuke Yamada

**Affiliations:** 1 Department of Pathology and Cell Biology, School of Medicine, University of Occupational and Environmental Health, Kitakyushu, 807–8555, Japan; 2 The First Department of Internal Medicine, School of Medicine, University of Occupational and Environmental Health, Kitakyushu, 807–8555, Japan; 3 Department of Cellular Pharmacology, Graduate School of Medicine, Hokkaido University, Sapporo, 060–8638, Japan; 4 Department of Biomolecular Function, Graduate School of Medical Science, Yamagata University, Yamagata, 990–9585, Japan; 5 Department of Occupational Pneumology, School of Medicine, University of Occupational and Environmental Health, Kitakyushu, 807–8555, Japan; 6 Shared-Use Research Center, School of Medicine, University of Occupational and Environmental Health, Kitakyushu, 807–8555, Japan; 7 Second Department of Surgery, School of Medicine, University of Occupational and Environmental Health, Kitakyushu, 807–8555, Japan; 8 Laboratory of Pathology, Hebei Cancer Institute, the Fourth Hospital of Hebei, Medical University, Jiankang Road 12, Shijiazhuang, 050011, Hebei, China; 9 Department of Thoracic Surgery, Saitama Cancer Center, Saitama, 362–0806, Japan; 10 The President Laboratory, School of Medicine, University of Occupational and Environmental Health, Kitakyushu, 807–8555, Japan; 11 Asahi-Matsumoto Hospital, Kitakyushu, 800–0242, Japan; 12 Department of Microbiology, University of Occupational and Environmental Health, Kitakyushu, 807–8555, Japan; 13 Laboratory of Pathology, Fukuoka Wajiro Hospital, Fukuoka, 811–0213, Japan; 14 Department of Pathology, Field of Oncology, Kagoshima University Graduate School of Medical and Dental Sciences, Kagoshima, 890–8544, Japan; 15 Institute of Pathology, Medical University of Graz, Graz, 8010, Austria; 16 Institute of Molecular Biosciences, University of Graz, Graz, 8010, Austria; University of Basque Country, SPAIN

## Abstract

**Background:**

Accumulating evidence has shown that methionine- and choline-deficient high fat (MCD+HF) diet induces the development of nonalcoholic fatty liver disease (NAFLD), in which elevated reactive oxygen species play a crucial role. We have reported that peroxiredoxin 4 (PRDX4), a unique secretory member of the PRDX antioxidant family, protects against NAFLD progression. However, the detailed mechanism and potential effects on the intestinal function still remain unclear.

**Methods & Results:**

Two weeks after feeding mice a MCD+HF diet, the livers of human PRDX4 transgenic (Tg) mice exhibited significant suppression in the development of NAFLD compared with wild-type (WT) mice. The serum thiobarbituric acid reactive substances levels were significantly lower in Tg mice. In contrast, the Tg small intestine with PRDX4 overexpression showed more suppressed shortening of total length and villi height, and more accumulation of lipid in the jejunum, along with lower levels of dihydroethidium binding. The enterocytes exhibited fewer apoptotic but more proliferating cells, and inflammation was reduced in the mucosa. Furthermore, the small intestine of Tg mice had significantly higher expression of cholesterol absorption-regulatory factors, including liver X receptor-α, but lower expression of microsomal triglyceride-transfer protein.

**Conclusion:**

Our present data provide the first evidence of the beneficial effects of PRDX4 on intestinal function in the reduction of the severity of NAFLD, by ameliorating oxidative stress-induced local and systemic injury. We can suggest that both liver and intestine are spared, to some degree, by the antioxidant properties of PRDX4.

## Introduction

Nonalcoholic fatty liver disease (NAFLD) is an umbrella term covering a broad spectrum of clinicopathological presentations, ranging from hepatic isolated steatosis to nonalcoholic steatohepatitis (NASH), a more severe form usually leading to end-stage liver diseases, including cirrhosis and/or hepatocellular carcinoma [1–3]. NAFLD is defined as the massive and ectopic accumulation of triglycerides (TG) in individuals who do not consume a large amount of alcohol (< 20 g ethanol/day), and it has been estimated to affect up to 30% of people in developed countries and more than 80% of obese people worldwide [1,4]. Indeed, NAFLD is tightly correlated with the development of metabolic syndrome, manifesting as obesity, dyslipidemia, type 2 diabetes mellitus (T2DM) and/or atherosclerosis [3,5,6]. However, much less is known regarding the systemic mechanisms underlying the transition from simple steatosis to NASH, even though many studies, including our own, have revealed that oxidative stress and endoplasmic reticulum (ER) stress appear to be strongly involved in these processes [6–9]. According to the well-known ‘two-hit’ hypothesis of NASH, the second hits include oxidative stressors and accumulation of reactive oxygen species (ROS) that overwhelm endogenous hepatocyte survival mechanisms, resulting in the development of NASH and cirrhosis [10,11]. However, most reports have only focused on the pathophysiology and molecular mechanism(s) in the target organ, the liver.

The small intestine, which has an extensive surface area and is also the first organ to encounter nutrients, may also play a critical role in metabolic disorders. In fact, there is a growing body of evidence suggesting that it has key functions in the etiology of obesity, insulin resistance and/or NAFLD, since it not only serves as a gatekeeper at the pathophysiological interface between the body and diet, but also has a central role in the efficient absorption and metabolic processing of nutrients [12,13]. Moreover, since the enterocytes in the small intestine are responsible for sensing the luminal contents modulated by the diet, especially a high-fat (HF) diet, the intestine can show an expression of various signaling molecules, including inflammatory cytokines/mediators and/or nuclear receptors, such as liver X receptors (LXRs), and subsequently, the liver, skeletal muscle and adipose tissue respond by modulating their metabolism to maintain homeostatic control [13]. It has previously been reported that the increased permeability caused by disruption of intercellular tight junctions is involved in the pathogenesis of NASH via the translocation of either intact bacteria or microbial cell components, i.e., lipopolysaccharide, into the circulation [14,15]. To clarify the intestinal function during the development of NAFLD, an appropriate animal model must be established. Although there are a few rodent models of NAFLD, representing one aspect of human metabolic syndrome, we planned to use a HF diet in addition to a classic methionine- and choline-deficient diet (MCD), to generate a MCD+HF diet (fat: 60%) [6,16,17], because mice fed only a MCD diet do not recapitulate the full spectrum of metabolic syndrome, manifesting only severe body weight loss and hypolipidemia [6,18]. To the best of our knowledge, no previous reports of experimental animals fed a MCD+HF diet have described the detailed histopathological and molecular features of the small intestine, and only a few studies have examined the relationship between oxidative stress and, both hepatic and intestinal function.

A newly identified family of antioxidant proteins, designated peroxiredoxin (PRDX), is ubiquitously synthesized and regulates the hydrogen peroxide levels by using thioredoxin as an electron donor [19–21]. The PRDX family includes at least six distinct PRDX genes that are expressed in mammals. Unlike the intracellular localization of other family members, PRDX4 is the only known secretory form [19,22,23] and protects against oxidative damage by scavenging ROS in both the intracellular compartments (especially, ER) and the extracellular space [5,6,23,24]. PRDX4 has an additional N-terminal region, which is unique to this enzyme, following a signal peptide that allows translocation across the ER membrane and is thereafter cleaved off [19]. We have recently established human PRDX4 (hPRDX4) transgenic (Tg) mice on a C57BL/6 background [5,6,25,26] and investigated the diverse protective roles of PRDX4 against a nongenetic mouse model of NASH and insulin resistance by feeding the mice a high-fructose diet after injecting a relatively low dose of streptozotocin [6]. It is conceivable that PRDX4 might help to protect against NAFLD and/or T2DM, and subsequently, metabolic syndrome, by reducing oxidative stress and synergistically suppressing steatosis, inflammatory reactions, and/or apoptotic activity, especially in the liver [6]. Despite these previous findings, much remains unknown regarding the pathophysiological relevance of PRDX4 in the intestinal functions during the progression of NAFLD.

In this study, we performed the first evaluation of the roles of PRDX4 in the pathogenesis of NAFLD, focusing on the small intestine. Using a MCD+HF diet-induced NAFLD model in Tg and control wild-type (WT) mice has allowed us to obtain novel insights into the protective effects of PRDX4, especially from the viewpoints of intestinal function. The present data indicate that the antioxidant and protective properties of PRDX4 can spare both liver and intestine to some degree, and beneficially affect intestinal function in the suppression of the severity of NAFLD, by reducing local and systemic oxidative stressors.

## Materials and Methods

### Construction of Human PRDX4 (hPRDX4) Transgenic Mice (Tg)

The primers for *hPRDX4* were designed based on a published sequence (Genebank accession no. NM_006406), and *hPRDX4* cDNA was amplified by reverse transcription–polymerase chain reaction (RT-PCR) and cloned into the pGEM-T easy vector system (Invitrogen, Life Technologies Japan Ltd., Tokyo, Japan), as described previously [6]. The entire nucleic acid sequence, containing a 0.6-kb cytomegalovirus (CMV) enhancer/promoter, the 0.8-kb *hPRDX4* cDNA, and the 0.2-kb bovine growth hormone polyadenylation (BGHPA) sequence, was microinjected into the male pronuclei of one-cell C57BL/6 mouse embryos using standard transgenic techniques to generate Tg mice [5,6,25]. The CMV enhancer/promoter shows extensive cross-talk with other promoters because it contains many transcription factor binding sites [5,6,27]. In contrast, as it is not a tissue-specific promoter, the protein expression of the *hPRDX4* transgene will be affected by its site of integration into the mouse genome. C57BL/6 mice (Charles River Laboratories, Yokohama, Japan) were used as the control WT mice in this study.

### Animals and Establishment of NAFLD

Experiments were performed using eight-week-old male WT and Tg mice weighing approximately 20 g, which were maintained in a temperature- and light-controlled facility with free access to water. Mice were fed an MCD+HF diet (60% fat; KBT Oriental Corporation, Saga, Japan) for two weeks to generate NAFLD, as described previously [6,16]. Animals were killed in a fed state at two weeks by intraperitoneal anesthetization with an overdose of ketamine (100 mg/kg) (Daiichi Sankyo Co., Tokyo, Japan) and medetomidine (2 mg/kg) (Meiji Yakuhin Co., Tokyo, Japan), and tissues, including the liver and small intestine, were excised. Serum samples were collected after removing blood cells and clotting factors from blood, which was taken from the axillary vein and artery. The body weight (BW) and food intake in one day were examined at day 0, 3, 6, 9, 12 and 14 in a fed state, and, the total food intake and organ weight were measured when the mice were killed. Food consumption was determined using metabolic cages obtained from SUGIYAMA-GEN CO., Ltd. (Tokyo, Japan).

### Ethics

All protocols were approved by the Ethics Committee of Animal Care and Experimentation, University of Occupational and Environmental Health, Japan, and were performed according to the Institutional Guidelines for Animal Experiments and the Law (no. 105) and Notification (no. 6) of the Japanese Government. The study also conforms to the Guide for the Care and Use of Laboratory Animals published by the US National Institutes of Health (NIH Publication No. 85–23, revised 1996).

### hPRDX4 Enzyme-Linked Immunosorbent Assays (ELISA)

The serum hPRDX4 levels at two weeks after starting the MCD+HF diet were measured using commercial ELISAs (hPRDX4: Abnova, Taipei, Taiwan).

### Measurement of Thiobarbituric Acid Reactive Substances (TBARS) Levels

We measured the serum TBARS levels using a TBARS Assay Kit (Cayman Chemical Company, Ann Arbor, MI, USA). The results are expressed as nmol malondialdehyde (MDA)/mg low-density lipoprotein (LDL) protein.

### Assessment of the Intracellular O_2_^-^ Levels Using Dihydroethidium (DHE) Staining

*In situ* staining of O_2_^-^ was performed using frozen mouse intestine and liver sections in order to determine the ROS/oxidative stress in enterocytes and hepatocytes [28]. Fresh tissue samples from the MCD+HF-induced NAFLD model mice were immediately frozen and stored at –80°C. Frozen tissue sections (6 μm thickness) were prepared for *in situ* imaging of O_2_^-^ with the DHE fluorescent dye (Molecular Probes, Oregon, USA). DHE concentration was finally 5 μM. The oxidative fluorescent dye, DHE, was freely permeable to cells, and in the presence of O_2_^-^ was oxidized to ethidium bromide (EtBr), which was trapped by intercalation with DNA. EtBr was excited at 488 nm with an emission spectrum of 610 nm. After staining, we quantified the number of cell nuclei (*red-stained*) positive for increased DHE associated fluorescence in 10 randomly selected fields per section (original magnification: × 400), as described previously [6,29].

### Histology and Immunohistochemistry (IHC)

A rabbit anti-human polyclonal antibody (1:500; Affinity BioReagents, Golden, CO, USA) was used to detect hPRDX4 [5,6,25]. Furthermore, we confirmed the IHC findings of PRDX4 using an original rabbit anti-rat PRDX4 antibody generated against the recombinant PRDX4 protein, as described previously [30]. To our knowledge, there are no specific antibodies commercially available for endogenous mouse PRDX4 (mPRDX4), and in fact, the amino acid sequence of mPRDX4 shows very high homology to that of hPRDX4 (> 89%), as determined by the Basic Local Alignment Search Tool (BLAST; National Center for Biotechnology Information, U.S. National Library of Medicine, Bethesda, MD, USA) [6].

For histological analyses of the liver and intestine, images of hematoxylin and eosin (H&E), oil red-O, Alcian blue, IHC or immunofluorescence (IF) sections were captured and quantified using the NanoZoomer Digital Pathology Virtual Slide Viewer software program (Hamamatsu Photonics Corp., Hamamatsu, Japan) [5,6,25,29]. To evaluate the degree of lipid accumulation (steatosis score for the liver; and lipid accumulation score for the intestine), we performed oil red-O staining using paraffin-embedded liver/intestine sections fixed in osmium tetroxide [5,6,18] and frozen liver/intestine sections, and categorized the tissues into four grades, as follows [6,18]: no lipid droplets (score = 0); lipid droplets in <33% of hepatocytes/enterocytes (score = 1); lipid droplets 33–66% of hepatocytes/enterocytes (score = 2) and lipid droplets in >66% of hepatocytes/enterocytes (score = 3).

To evaluate the severity of NAFLD, we determined the intensity of inflammation using an anti-mouse Mac-2 monoclonal antibody (1:500; Cedarlane Laboratories Ltd., Burlington, Ontario, Canada) or a polyclonal rabbit anti-human CD3 antibody (1:1; DAKO Cytomation Co., Tokyo, Japan), as described previously [5,6,25,29]. We counted the number of positive macrophages or T lymphocytes in 10 randomly selected fields per liver or intestine section (original magnification: × 400). The NAFLD liver tissues were then classified into four (inflammation score) grades, as follows [6,18]: no inflammation (score = 0); <10 inflammatory foci, each consisting of >5 inflammatory cells (score = 1); ≥10 inflammatory foci (score = 2) or uncountable diffuse or fused inflammatory foci (score = 3). Furthermore, the degree of liver cell ballooning injury (ballooning score) was classified into three grades as follows: none (score = 0); few balloon cells (score = 1) or many balloon cells/prominent ballooning (score = 2). Liver fibrosis was quantified by Masson’s trichrome staining. For IHC and IF studies, we examined at least one section from each of 10 mice per experimental group.

All histological and IHC/IF slides were evaluated by two independent observers who were blinded to the physical outcome and other biological and pathological data for each sample. In case of disagreement, a consensus score was determined by a third board-certified pathologist.

### Terminal Deoxynucleotidyl Transferase End-Labeling (TUNEL) and 5’-Bromo-2’-Deoxyuridine (BrdU) Staining

TUNEL assays were performed using an *In Situ* Cell Death (Apoptosis) Detection Kit, POD (Roche Applied Science, Mannheim, Germany) [6,29,31]. Next, to label proliferating enterocytes in the crypts two weeks after beginning the MCD+HF feeding, BrdU (50 mg/kg body weight; Sigma, St. Louis, MO, USA) was subcutaneously injected 1 hr before sacrifice. BrdU-incorporated cells were detected by IHC using a monoclonal mouse anti-BrdU antibody (Roche Applied Science, Lewes, UK) [29]. For quantitative analyses, we counted TUNEL^+^ or BrdU^+^ enterocytes (*brown-stained*) in 10 randomly selected fields in the crypts per section (original magnification: × 400).

### Analyses of Lipid Contents from Liver and Small Intestine, and Hepatic Injury

To examine the hepatic and intestinal lipid profiles, each snap frozen tissue (30 mg) was homogenized and extracted with chloroform-methanol (2/1 v/v), as described previously [6,29]. The organic phase was dried and resolubilized in 2-propanol. Then, the triglyceride (TG), free fatty acid (FFA) and cholesteryl ester (CE) contents were determined using commercial assay kits (Wako Pure Chemical Co., Osaka, Japan).

The serum levels of hepatic injury-related enzymes, including alanine aminotransferase (ALT) and aspartate aminotransferase (AST), were also measured using commercial assay kits (Wako Pure Chemical Co.) [6,18].

### High-Performance Liquid Chromatography (HPLC) Analysis of Lipoproteins

After feeding the mice MCD+HF for two weeks, they were fasted for 7 hr, and blood was collected from the axillary artery into a micro-tube containing 5 μl of 0.5 mol/L ethylenediaminetetraacetic acid (EDTA). Samples were centrifuged for 12 min at 5500 ×*g* at 4°C and the resulting serum was stored at –80°C until it was assayed. Lipoproteins were analyzed by HPLC using molecular sieve columns (Skylight Biotech, Inc. Akita, Japan), as described previously [18,32].

### RT-PCR and Real-Time RT-PCR

RT-PCR and real-time RT-PCR were used to analyze the gene expression in the liver and small intestine. The primers and probes used for each gene are listed in S1 Table. The relative expression levels of each gene were normalized to those of *glyceraldehyde 3-phosphate dehydrogenase* (*GAPDH*) and 18*S* ribosomal RNA (rRNA) using random primers, as reported previously [6,18,29].

### Western Blotting

Liver and intestine protein samples were separated by electrophoresis on 10% SDS-PAGE gels and transferred onto Immun-Blot PVDF membranes (Bio-Rad Laboratories, K.K., Tokyo, Japan). The membranes were then incubated with a rabbit anti-human polyclonal hPRDX4 antibody (1:3,000; Affinity BioReagents), protein disulfide isomerase (PDI) (1:500; Santa Cruz Biotechnology, Dallas, USA), microsomal TG-transfer protein (MTP) (1:10,000; BD Transduction Laboratories, San Jose, USA), LXR-α (1:500; Santa Cruz Biotechnology), apolipoprotein B (ApoB) (1:1,000; Millipore, Tamecula, USA), ApoE (1:1,000; Abcam, Eugene, USA) and mouse anti-chicken monoclonal β-actin antibodies (1:1,000; Santa Cruz Biotechnology).

### MTP Activity Assay

The MTP activities of the hepatic or intestinal microsomes were measured using a commercial kit (Chylos Inc., Woodbury, NY, USA) according to the manufacturer’s instructions [33]. Hepatic or intestinal lysates were prepared from fresh tissues as described above and were immediately subjected to the reaction.

### Evaluation of the Intestinal Microbiota Using the 16S Ribosomal RNA Gene-Based Clone Library Method

Ten fecal samples were used, which were collected from WT (n = 5) and Tg (n = 5) mice two weeks after they were started on a MCD+HF diet. The fecal samples were collected in sterile tubes and immediately stored at 4°C, and were analyzed within seven days [34]. A stool sample (0.1 g) was suspended in 3 mL of DNA extraction buffer (100 mM Tris–HCl, pH 8.0, 200 mM sodium EDTA-2Na), and was vortexed for 30 seconds. After the treatment with an Astrason XL-2020 ultrasonic processor (Misonix, Farmingdale, NY, USA) for 15 seconds, the suspension was diluted 400 times with the DNA extraction buffer. The suspension was used for cell counting and for DNA extraction.

The feces suspension (100 mL) was combined with 900 mL of EtBr solution (100 mg/mL in 0.1 M phosphate pH 8.5 buffer, 5% NaCl, 0.5 mM sodium EDTA) at room temperature for 10 min. In order to collect bacterial cells, the combination (1.0 mL) was then filtered through a pore-filter of 0.2 mm (Millipore, Bedford, MA, USA) and was rinsed once with 3.0 mL filtered water. Filtered immersion oil on non-fluorescent slide glasses was used to fix the filters. Bacteria-shaped objects were observed using an Olympus BX50 microscope (Olympus Optical, Tokyo, Japan) and were counted from 30 randomly selected fields per slide. The number of bacteria per gram of fecal sample was calculated.

A feces suspension (900 mL) was mixed with 100 mL of 30% sodium dodecyl sulfate (SDS) solution and approximately 0.3 g of a mixture of glass beads that consisted of equal weights of 0.1 mm- and 1 mm-diameter beads in a 2.5 mL polypropylene tube. The mixtures were strongly shaken by a Micro Smash MS-100 apparatus (Tomy Seiko Co., Ltd., Tokyo, Japan) for 5 min at 4500 rpm. The supernatant was collected by centrifugation at 20,000 ×*g* for 5 min at room temperature, and was transferred to a fresh tube. This DNA extraction procedure was carried out three times. The three supernatants were combined and extracted with an equal volume of phenol–chloroform–isoamyl alcohol (25:24:1, v/v). The DNA in the aqueous phases was concentrated to 30 mL of TE buffer using a Montage PCR centrifugal filter device (Millipore, Bedford, MA, USA).

The clone library construction and nucleotide sequencing analysis was performed as described previously [34]. Using the extracted DNA as a template, a fragment of the 16*S* ribosomal ribonucleic acid (rRNA) gene (550 bp) was amplified with universal primers, E341F (5’-CCTACGGGAGGCAGCAG-3’) and E907R (5’-CCGTCAATTCMTTTRAGTTT-3’) [34]. The PCR amplification was performed with AmpliTaq Gold DNA polymerase using a GeneAmp PCR system 9700 thermocycler (Applied Biosystems, Foster City, CA, U.S.A.). The cycling conditions were 96°C for 5 min, followed by 30 cycles of 96°C for 30 seconds, 53°C for 30 seconds and 72°C for 1 min, with a final elongation step at 72°C for 7 min. The PCR products were confirmed with a 2.0% agarose gel electrophoresis.

The PCR products were cloned into *Escherichia coli* TOP10 cells using a TOPO TA Cloning kit (Invitrogen, Carlsbad, CA, USA). For the nucleotide sequencing analysis, a total of 96 white colonies from each sample were randomly selected. To prepare a template for the sequencing analysis, the inserted PCR product was amplified with M13 forward and reverse primers. An aliquot (1 mL) was used for the sequencing reaction after primers and deoxynucleoside triphosphate (dNTP) were eliminated from the PCR mixture with ExoSAP-IT (USB, Cleveland, OH, USA). Sequencing reactions were carried out with the M13 forward primer and BigDye Terminator v3.1 Cycles Sequencing Kit (Applied Biosystems). The nucleotide sequences were determined by a 3130xl Genetic Analyzer (Applied Biosystems).

Quality checking and the trimming of nucleotide sequences were performed by the DNASIS Pro software program (Hitachi Software Engineering, Tokyo, Japan). Highly accurate sequences selected by the PHRED quality value were trimmed from the primer and vector regions. In order to exclude chimeric sequences, the nucleotide sequences were analyzed with the Bellerophone chimera check program. A homology search was performed with the BLAST algorithm, using an in-house database based on the sequences of type strains obtained from the Ribosomal Database Project (http://rdp.cme.msu.edu/).

### Statistical Analysis

The results are expressed as the means ± SE. Significant differences were analyzed using Student’s *t*-test, Welch’s *t*-test or the one-way ANOVA (analysis of variance), where appropriate. In all cases when ANOVA methodology was employed, Tukey’s multiple comparison *post-hoc* test was used. Values of *p* < 0.05 were considered to be statistically significant.

## Results

### Expression of PRDX4 in Mice of MCD+HF Diet-Induced NAFLD Model

**Fig 1:**
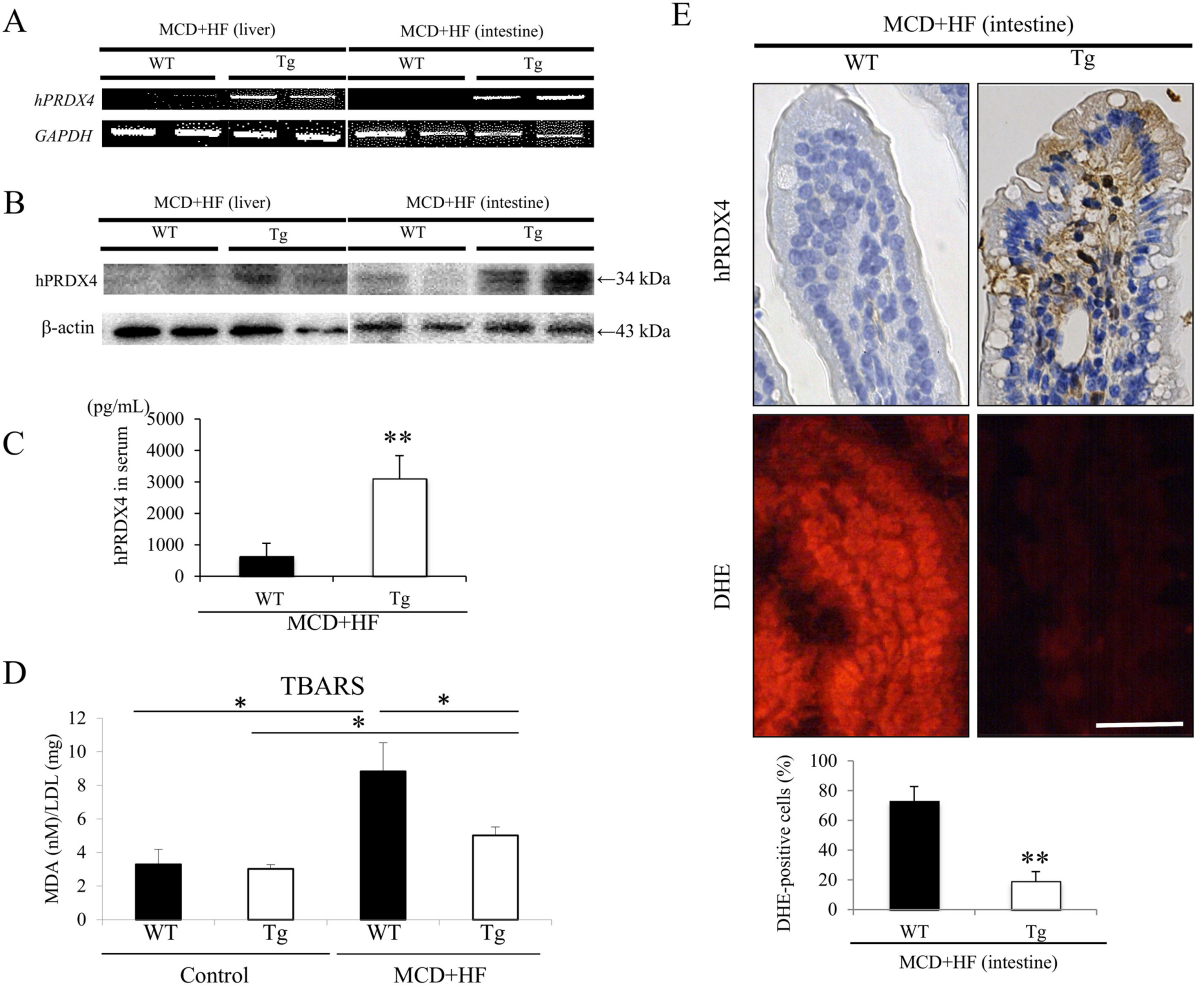
Analyses of the expression of hPRDX4 mRNA and protein, and oxidative stress markers in the mice. **A)** RT-PCR showed that the expression levels of hPRDX4 from both the liver and small intestine of MCD+HF-fed model Tg mice were markedly elevated, but this was not the case for those from the WT mice (n = 10 mice per group). **B)** A Western blot analysis revealed significantly increased hepatic and intestinal total PRDX4 protein expression in MCD+HF-fed model Tg mice (n = 10 mice per group). Very faint bands were detected in model WT mice, indicating weak cross-reactivity of the antibody for mPRDX4, as described previously [5,6,25]. **C)** ELISAs confirmed that the serum circulatory hPRDX4 levels were markedly higher in MCD+HF-fed model Tg mice (n = 10 mice per group) than those in the WT mice (**p < 0.01). **D)** The serum TBARS level expressed as nmol MDA/mg LDL protein was significantly more suppressed in model Tg mice than in model WT mice after 2 weeks of the MCD+HF diet (n = 10 mice per group). The TBARS levels of MCD+HF model mice were significantly higher than those of control mice (n = 5 mice per group). *p < 0.05. **E)** IF showed that the numbers of increased DHE associated fluorescence (*red-stained*) within enterocytes were significantly higher in the intestines from model WT mice than in those from Tg mice (MCD+HF, n = 10 mice per group). Consistently, IHC revealed that hPRDX4 was detected in a number of enterocytes throughout the small intestine of model Tg mice, but not in those from model WT mice **p < 0.01. Original magnification: × 400. *Bar* = 50 μm. The values are the means ± SE and were normalized to the *GAPDH* expression (RT-PCR) in A, and for β-actin expression in B (Western blotting). *p < 0.05, **p < 0.01.

As reported previously, the mRNA and protein expression levels of hPRDX4 in various tissues from untreated Tg mice have been detected by RT-PCR and Western blotting analyses, respectively [5,6,25]. PCR clearly showed that *hPRDX4* was only detected in the liver and small intestine of Tg mice, but not in those of WT mice (Fig 1A and S1A Fig). Additionally, its expression was significantly higher in Tg mice used to generate the MCD+HF-induced NAFLD model than in non-induced Tg mice (data not shown). Interestingly, real-time RT-PCR revealed that the gene expression levels of most of the *mPRDXs* examined, including endogenous *mPRDX4*, were significantly lower in the livers and intestines from model Tg mice, than those from WT mice (S1B Fig). In the mice that developed NAFLD, a Western blot analysis (Fig 1B) confirmed that the final levels of total PRDX4 protein was highly expressed throughout the liver (significantly 2.1-fold more elevated) and intestine (significantly 5.4-fold more elevated) of the Tg mice, compared to the WT mice. To determine the level of circulating hPRDX4 protein, we performed ELISA using serum samples obtained after inducing the MCD+HF mouse model. These tests also confirmed that Tg mice had significantly elevated PRDX4 levels compared with WT mice (WT 627.3 ± 423.4 pg/mL vs. Tg 3106.5 ± 727.8 pg/mL; *p* < 0.01) (Fig 1C). A Western blotting analysis (Fig 1B) demonstrated that the livers of WT mice had a faint band corresponding to the anti-hPRDX4 antibody, indicating cross-reactivity of the antibodies against hPRDX4 to mPRDX4, as previously described [5,6,25].

### Levels of Serum TBARS and Tissue DHE in WT and Tg Mice Before and After the Development of MCD+HF Diet-Induced NAFLD

Because PRDX4 has significant anti-oxidant activity, we examined the serum levels of an oxidative stress marker, TBARS [5,6]. The serum TBARS levels were significantly more suppressed in Tg mice than in WT mice after two weeks on the MCD+HF diet (WT 8.83 ± 1.70 nmol MDA/mg LDL protein vs. Tg 5.02 ± 0.50 nmol MDA/mg LDL protein, *p* < 0.05) (Fig 1D)), and this marker was not significantly different between control (before starting diet) WT and Tg mice even at significantly lower levels (WT 3.29 ± 0.90 nmol MDA/mg LDL protein vs. Tg 3.02 ± 0.25 nmol MDA/mg LDL protein). Next, we used IF to determine the increased DHE associated fluorescence as another marker of the *in situ* production of ROS. The numbers of DHE-positive intestinal enterocytes and hepatocytes were very small in untreated WT and Tg mice (data not shown), but their numbers were significantly increased by feeding them the MCD+HF diet. Moreover, there were significantly fewer enterocytes (Fig 1E) and hepatocytes (S2 Fig) positive for DHE in the nuclei of Tg mice than in WT mice at two weeks after the MCD+HF diet (enterocytes: WT 72.7 ± 10.0 vs. Tg 18.9 ± 6.6; *p* < 0.01) (hepatocytes: WT 65.4 ± 9.6 vs. Tg 13.2 ± 2.2; *p* < 0.01). IHC confirmed that hPRDX4 was expressed in the corresponding enterocytes (Fig 1E) and hepatocytes (S2 Fig) from model Tg mice, but not in those from model WT mice (Fig 1E).

### Comparison of Metabolic Parameters between WT and Tg Mice Before and After the Development of MCD+HF Diet-Induced NAFLD

**Fig 2:**
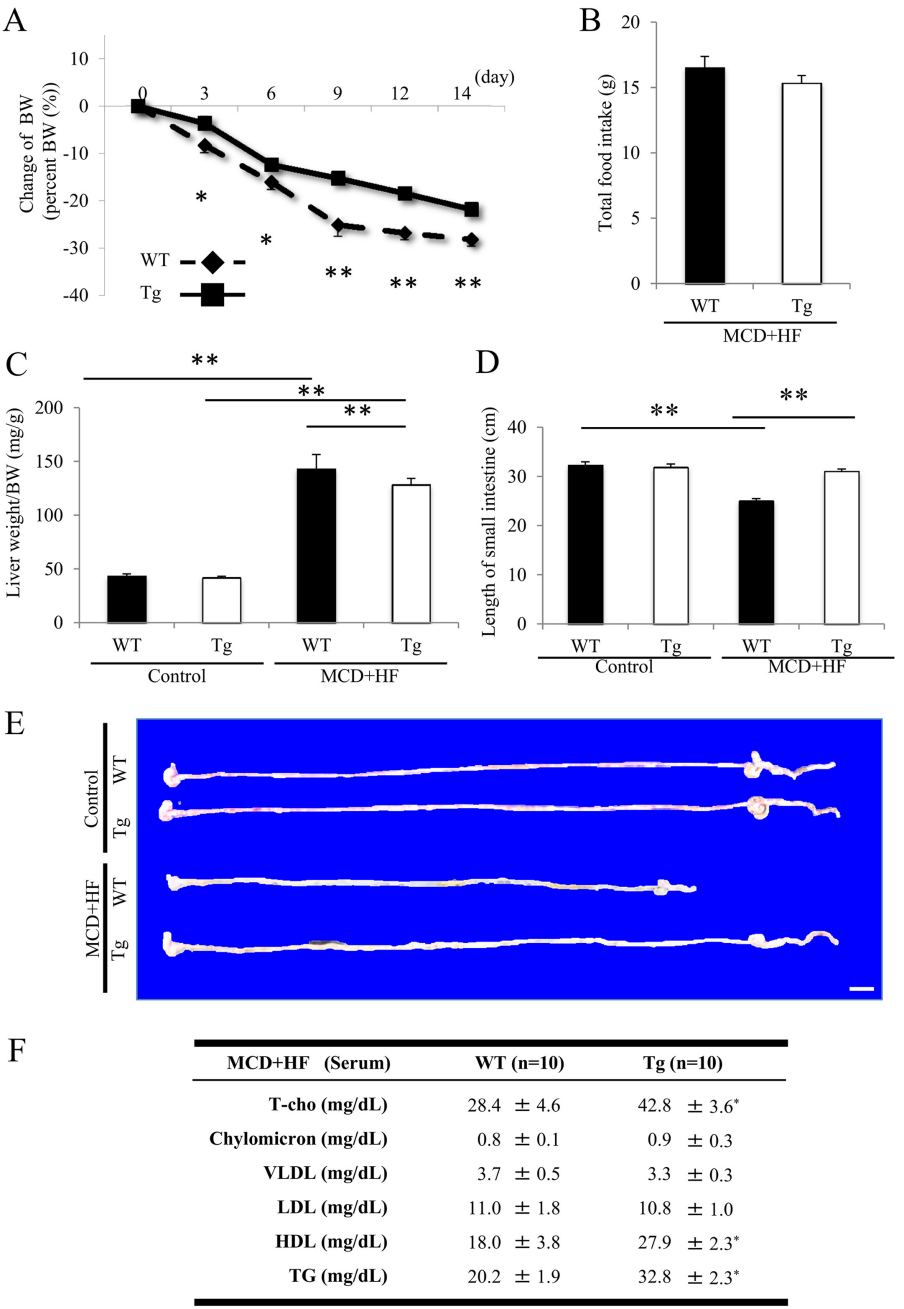
Comparison of the metabolic parameters and morphometry of the small intestine between model WT and Tg mice following induction of the MCD+HF feeding. **A**) The present model was not overtly obese, but the BW loss was significantly smaller in model Tg mice 2 weeks after MCD+HF feeding than that in model WT mice (*p* < 0.05 to 0.01) (MCD+HF, n = 10 mice per group). **B)** The food consumption was not significantly different between the 2 groups of mice in the entire experimental period for 2 weeks after MCD+HF feeding (n = 10 mice per group). **C)** Neither significant changes nor differences in gross appearances of the liver under basal conditions were noted between control Tg and WT mice (n = 5 mice per group). However, after 2 weeks of MCD+HF feeding, the mouse liver/BW ratios were significantly more increased (*p* < 0.01), but those in model Tg mice were significantly more suppressed than those in model WT mice (*p* < 0.01) (MCD+HF, n = 10 mice per group). **D,E)** Macroscopic observation revealed no significant difference in the gross appearance of the small intestine between control Tg and WT mice (n = 5 mice per group). The intestinal tissues from Tg mice showed no remarkable change in their gross appearance before and after the development of the MCD+HF dietary NASH model, whereas those from model WT mice demonstrated significantly shortened intestinal lengths compared with control WT mice (*p* < 0.01). *Bar* = 10 mm. **F)** The serum cholesterol level was determined by HPLC in mice fed the MCD+HF for 2 weeks (n = 10 mice per group). Both model WT and Tg mice developed modest hypolipidemia with variably decreased levels of T-cho, chylomicrons, VLDL, LDL and TG. However, the serum levels of T-cho, chylomicrons, HDL and TG were significantly higher in Tg mice than in WT mice. There was no significant difference in the VLDL or LDL levels between the 2 groups. The values are the means ± SE. *p < 0.05, **p < 0.01, ***p < 0.001.

The BW evaluations revealed that the present NAFLD model was not overtly obese, but the BW loss was significantly smaller in the model Tg mice the first two weeks after the MCD+HF diet, than that in the WT mice (Fig 2A; *p* < 0.05 to 0.01) (day 14: WT –26.5 ± 1.5% vs. Tg –23.5 ± 1.1%; *p* < 0.01). The food consumption was slightly decreased after the MCD+HF diet compared to before the diet in both the WT and Tg mice (data not shown), but the difference was not significant both in one day (data not shown) and in the entire experimental period (total 2 weeks) (Fig 2B) between the two groups of mice. Morphological studies revealed neither significant change nor difference in the gross appearance of the liver (Fig 2C) or small intestine (Fig 2D and 2E) under basal conditions between untreated Tg and WT mice, as described previously [5,6,25]. However, after two weeks of the MCD+HF diet, the mouse liver/BW ratios were significantly more increased (Fig 2C; *p* < 0.01), while those in the model Tg mice were significantly more suppressed than those in the model WT mice (Fig 2C; *p* < 0.01). In addition, the intestinal tissues from Tg mice showed no remarkable change in their gross appearance before or after the development of the MCD+HF diet-induced NAFLD model, whereas the model WT mice demonstrated significantly shortened intestinal lengths compared with the control WT mice (MCD+HF: WT 25.0 ± 0.5 cm vs. Tg 31.0 ± 0.5 cm; *p* < 0.01) (Control: WT 32.3 ± 0.7 cm vs. Tg 31.9 ± 0.7 cm) (Fig 2D and 2E).

We determined the serum cholesterol profiles by HPLC in mice fed the MCD+HF diet for two weeks. Both WT and Tg model mice developed modest hypolipidemia with variably low levels of total cholesterol (T-cho), chylomicrons, very low-density lipoprotein (VLDL), LDL and TG. However, the serum levels of T-cho, high-density lipoprotein (HDL) and TG were significantly higher in Tg mice than in WT mice (*p* < 0.05; Fig 2F). There was no significant difference in the chylomicron, VLDL or LDL levels between the two groups (Fig 2F).

### Histological and Morphometric Analyses of the WT and Tg Livers After the Development of MCD+HF Diet-Induced NAFLD

**Fig 3:**
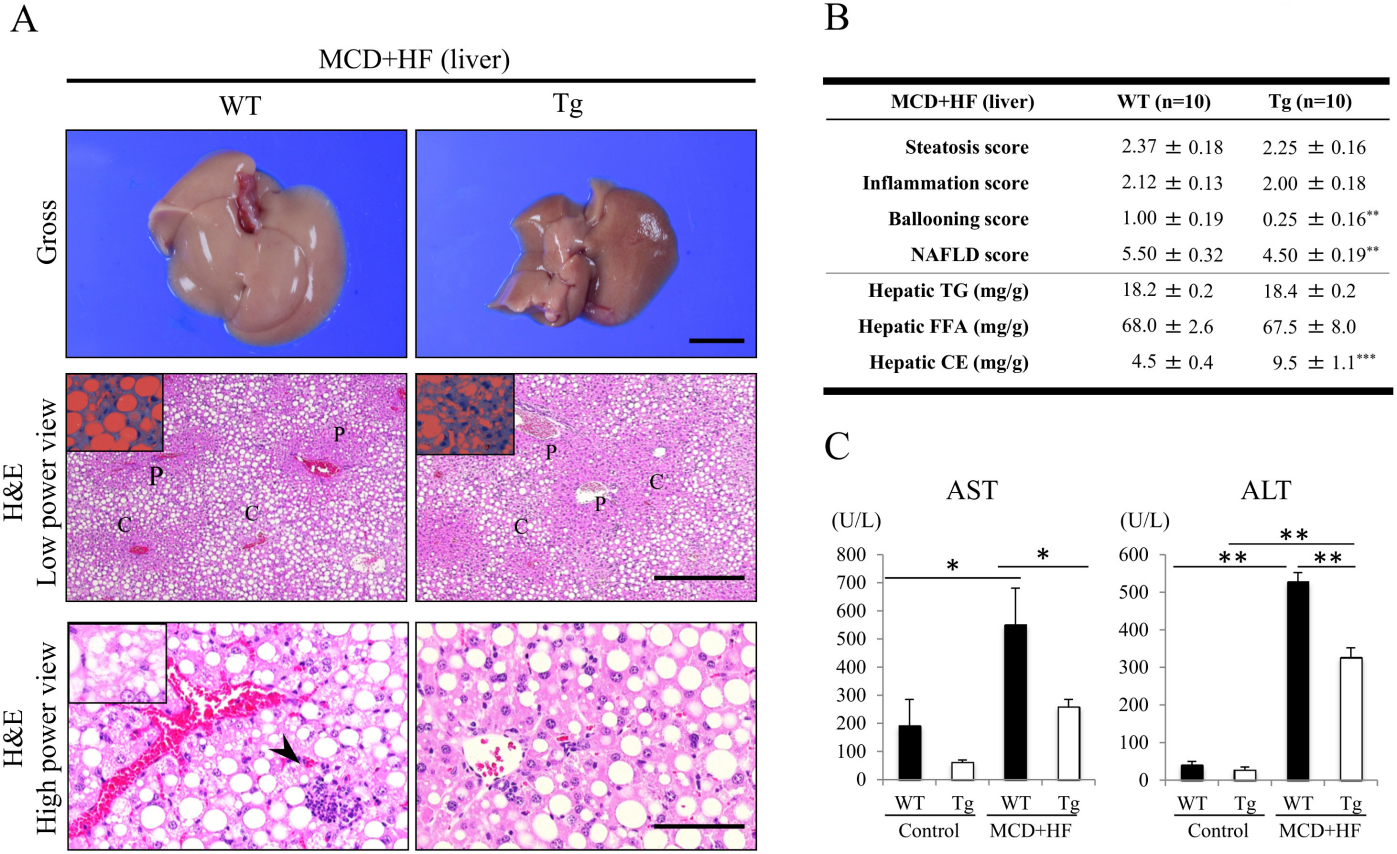
Histological and morphometric analyses of the liver of MCD+HF model mice. **A)** The gross appearance (upper) of the NAFLD livers from mice fed the MCD+HF for 2 weeks (n = 10 mice per group). The liver tissues from both types of model mice were variably pale and yellowish in color, indicative of lipid accumulation. Livers from WT mice were significantly enlarged. *Bar* = 5 mm. Oil-red O staining confirmed lipid accumulation in the both NAFLD livers (inset). Representative low- to high-power views of H&E-stained sections (middle and bottom) revealed that the WT liver had a tendency to contain more lipid droplets and inflammatory foci (arrowhead), than those of Tg mice, even though there was no significant histological difference. In contrast, hepatocyte ballooning was occasionally, but significantly, seen in the model WT liver (inset), but very rarely in the model Tg liver. The lipid droplets in the NAFLD livers of model mice were of varying size, indicating mixed microvesicular to macrovesicular steatosis. C = central vein and P = portal vein. Original magnification: × 40 (low) and × 200 (high). *Bars* = 400 μm (low) and 80 μm (high). **B)** Quantitative scoring of fat accumulation, inflammation and ballooning in the NAFLD livers of model WT and Tg mice (MCD+HF, n = 10 mice per group). The ballooning scores were significantly higher in the WT livers than in the Tg livers. Consequently, the NAFLD scores were also higher in the WT livers than in the Tg livers. The hepatic CE, but not TG and FFA, contents were significantly higher in model WT mice than in model Tg mice. **C)** The plasma AST and ALT levels were significantly higher in model WT mice than in control WT mice (control, n = 5 mice per group) and in model Tg mice (MCD+HF, n = 10 mice per group). The values are the means ± SE. *p < 0.05, **p < 0.01, ***p < 0.001.

After two weeks of the MCD+HF diet, the liver tissues from model mice were variably pale and yellowish in color (Fig 3A), indicating lipid accumulation, whereas those from WT mice were significantly enlarged in the gross findings (Fig 3A). H&E staining showed that the Tg liver had a tendency to contain fewer lipid droplets and inflammatory foci (steatosis score: 2.25 ± 0.16; inflammation score: 2.00 ± 0.18), than those of WT mice, even though there was no significant histological difference (steatosis score: 2.37 ± 0.18; inflammation score: 2.12 ± 0.13) (Fig 3A and 3B). IHC for Mac-2 revealed that the model Tg livers contained a significantly smaller number of infiltrating macrophages (Kupffer cells) than that in the WT mice (WT 9.3 ± 1.1 vs. Tg 3.7 ± 1.0; *p* < 0.05) (S3A Fig). CD3-staining also showed that there were fewer T lymphocytes in the model Tg liver than in the model WT liver (WT 42.1 ± 1.5 vs. Tg 34.4 ± 2.7; *p* < 0.05) (S3B Fig).

Similarly, real-time RT-PCR demonstrated that the expression levels of proinflammatory signaling molecules, including *tumor necrosis factor* (*TNF)-α*, *interleukin* (*IL)-1β*, *toll-like receptor* (*TLR*) *4*, and *nuclear factor κ-B1a* (*NFκ-B1a*) were significantly lower in the livers of model Tg mice than those in model WT mice (*p* < 0.05 or 0.01) (S3C Fig). Moreover, hepatocyte ballooning was occasionally, but significantly more often, seen in the model WT liver than in the model Tg liver (ballooning score: WT 1.00 ± 0.19 vs. Tg 0.25 ± 0.16; *p* < 0.01) (Fig 3A and 3B). The lipid droplets in the NAFLD livers of model mice were of varying size, indicating mixed microvesicular to macrovesicular steatosis (Fig 3A). In addition, oil red-O staining on the frozen sections confirmed hepatic lipid accumulation in both types of model mice (Fig 3A), as the hepatic TG and FFA contents in the two groups were substantially high (TG: WT 18.2 ± 0.2 mg/g vs. Tg 18.4 ± 0.2 mg/g) (FFA: WT 68.0 ± 2.6 mg/g vs. Tg 67.5 ± 8.0 mg/g) (Fig 3B). Perivenular and pericellular fibrosis, as determined by Masson’s trichrome staining, were rarely seen in either the model Tg or WT livers (data not shown).

The serum levels of both ALT and AST were significantly increased in model WT mice, compared to those in model Tg mice and control mice (Fig 3C), indicating greater hepatocyte cell death (apoptosis) [6]. The histopathological features, including the induction of macrovesicular steatosis by lipid accumulation, the ballooning of hepatocytes, and the presence of inflammation in the livers of model WT mice were very similar to those seen in human NAFLD. Correspondingly, the NAFLD scores from the model WT livers were significantly higher than those in the model Tg livers (WT 5.50 ± 0.32 vs. Tg 4.50 ± 0.19; *p* < 0.01) (Fig 3B).

### Hepatic Expression of PDI, MTP, LXR-α and Apolipoproteins in WT and Tg Mice after the Development of MCD+HF Diet-Induced NAFLD

**Fig 4:**
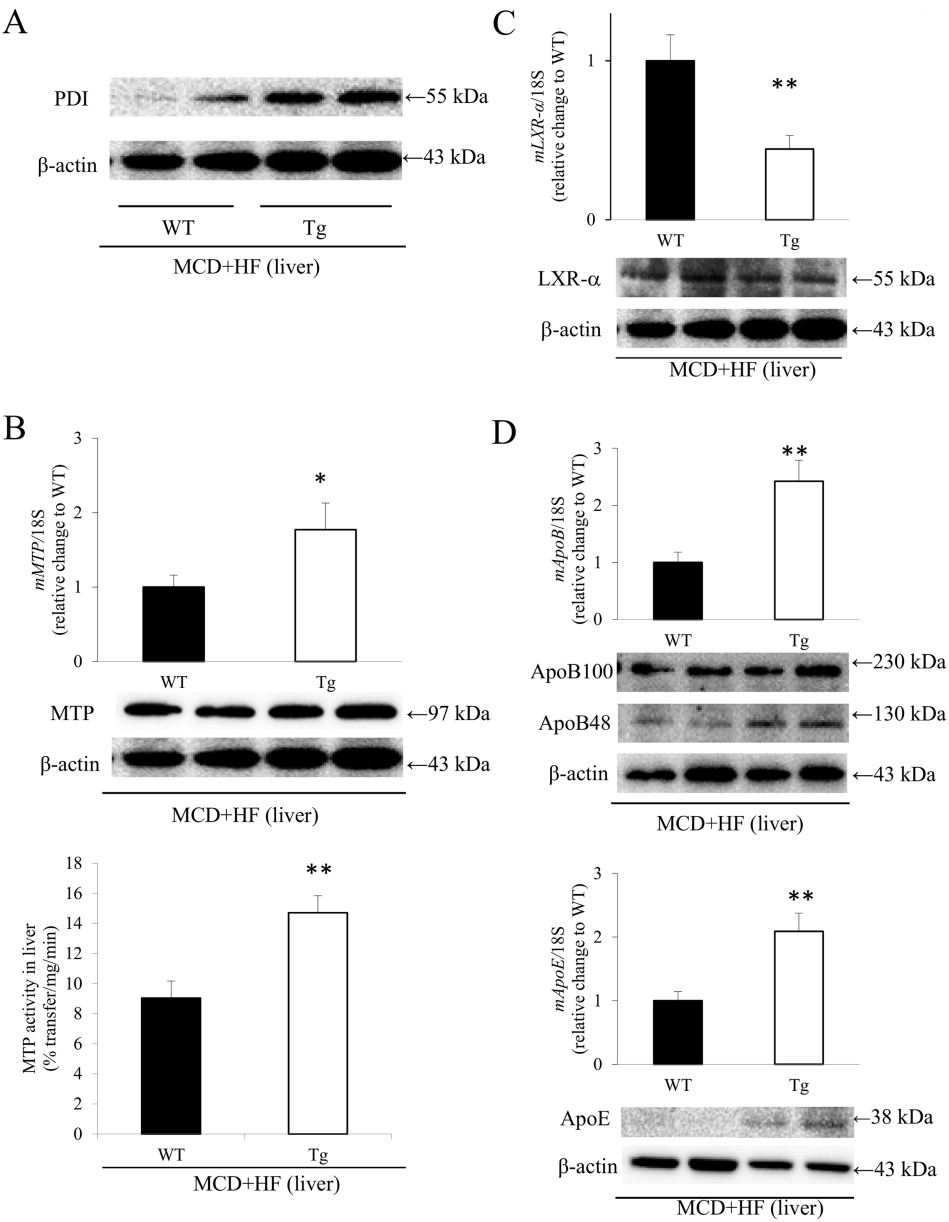
Analyses of the expression of PDI, MTP, LXR-α and apolipoproteins in the NAFLD livers of MCD+HF model mice. **A)** A Western blot analysis showed that the hepatic expression levels of PDI were significantly higher in model Tg mice than in model WT mice (MCD+HF, n = 10 mice per group). **B)** The real-time RT-PCR analyses (upper) and assays of lipid-transfer activity (lower) of MTP revealed that the hepatic *MTP* mRNA expression levels and activity were significantly higher in model Tg mice than in model WT mice, even though there was no significant difference in the hepatic MTP protein levels (middle) between the 2 groups of model mice (MCD+HF, n = 10 mice per group). **C)** In contrast, the hepatic *LXR-α* mRNA expression levels (upper) were significantly lower in model Tg mice than those in model WT mice, which was confirmed by a Western blot analysis (lower) of LXR-α (MCD+HF, n = 10 mice per group). **D)** The mRNA expression levels of *ApoB* and *ApoE* (upper) were significantly higher in the livers of model Tg mice than those in model WT mice, as confirmed by Western blot analysis (lower) of ApoB48 and 100 and ApoE (MCD+HF, n = 10 mice per group). The values are the means ± SE, and were normalized to the 18*S* rRNA expression (real-time RT-PCR) and β-actin expression (Western blotting). *p < 0.05, **p < 0.01.

Western blot analysis revealed that the hepatic expression levels of PDI were significantly higher in model Tg mice than in model WT mice (Fig 4A). In addition, real-time RT-PCR and an assay of the lipid-transfer activity of MTP showed that the hepatic *MTP* mRNA expression levels and activity were significantly higher in model Tg mice than in model WT mice (PCR: *p* < 0.05) (activity: WT 9.03 ± 1.13% transfer/mg/min vs. Tg 14.71 ± 1.14% transfer/mg/min; *p* < 0.01) (Fig 4B), even though there was no significant difference in the hepatic MTP protein levels between the two groups of model mice (Fig 4B).

In contrast, the hepatic *LXR-α mR*NA levels were significantly lower in model Tg mice than those in model WT mice (*p* < 0.05; Fig 4C), as confirmed by a Western blot analysis of LXR-α (Fig 4C). Correspondingly, real-time RT-PCR showed that the expression levels of *ApoB* and *ApoE* were significantly higher in the livers of model Tg mice than those in model WT mice (*p* < 0.01; Fig 4D), which was confirmed by a Western blot analysis of ApoB48 and 100, and ApoE (Fig 4D).

### Histological and IHC Analyses of WT and Tg Jejunum After the Development of the MCD+HF Diet-Induced NAFLD Model

**Fig 5:**
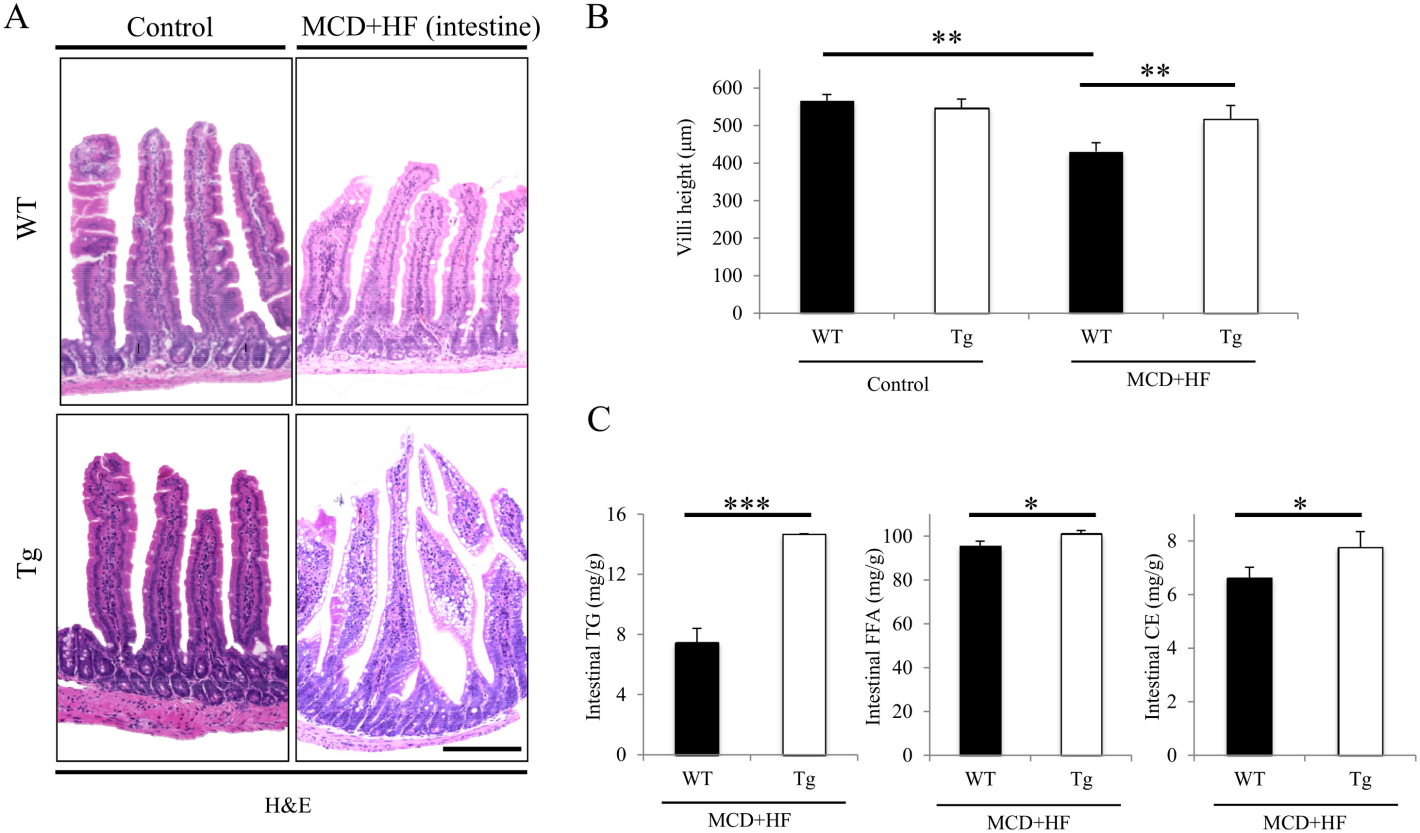
Histological and morphometric analyses of the small intestines of MCD+HF model mice. **A)** The histological appearance of the jejunum from control mice (n = 5 mice per group) and model mice fed the MCD+HF diet for 2 weeks (n = 10 mice per group). Representative low-power views of H&E-stained sections showed that the jejunal villi height (i.e., the absorption surface area) was significantly shortened in model WT mice compared with model Tg mice and normal control mice, whereas the lengths in the model Tg mice revealed significantly suppressed villi shortening. Original magnification: × 100 (low). *Bar* = 200 μm. **B)** The measurement of the jejunal villi height revealed significantly decreased shortening in model Tg mice, but significant shortened changes in model WT mice (MCD+HF, n = 10 mice per group), compared with the normal control groups (n = 5 mice per group). **C)** Quantitative analyses of the lipid accumulation in the intestines of model WT and Tg mice (MCD+HF, n = 10 mice per group). The intestinal TG, FFA and CE contents were significantly higher in model Tg mice than in model WT mice. The values are the means ± SE. *p < 0.05, **p < 0.01, ***p < 0.001.

Corresponding to the gross findings (Fig 2D and 2E), H&E staining revealed that the jejunal villi height (i.e., the absorption surface area) (Fig 5A and 5B) was significantly shortened in the model WT mice compared with the model Tg mice and the normal control mice, whereas the heights in the model Tg groups exhibited significantly suppressed villus shortening (MCD+HF: WT 431.0 ± 23.4 μm vs. Tg 517.1 ± 36.6 μm; *p* < 0.01) (Control: WT 565.8 ± 16.8 μm vs. Tg 546.4 ± 24.0 μm) (Fig 5A and 5B). The intestinal TG, FFA and CE contents in the model Tg intestine were significantly greater than those in model WT mice (TG: WT 7.5 ± 1.8 mg/g vs. Tg 14.6 ± 1.9 mg/g; *p* < 0.001) (FFA: WT 95.4 ± 2.3 mg/g vs. Tg 101.1 ± 1.5 mg/g; *p* < 0.05) (CE: WT 6.6 ± 0.5 mg/g vs. Tg 7.8 ± 0.2 mg/g; *p* < 0.05) (Fig 5C). Additionally, the model Tg intestine tended to contain more accumulation of small lipid droplets in the surface enterocytes (i.e., higher lipid accumulation score), than the model WT intestine (data not shown), however, no significant difference was evident. Positive oil red-O staining, but negative Alcian blue staining, confirmed the intestinal lipid accumulation, especially in the model Tg mice (data not shown).

### Analyses of the Apoptotic and Proliferative Activities in the WT and Tg Jejunum After the Development of the MCD+HF Diet-Induced NAFLD Model

**Fig 6:**
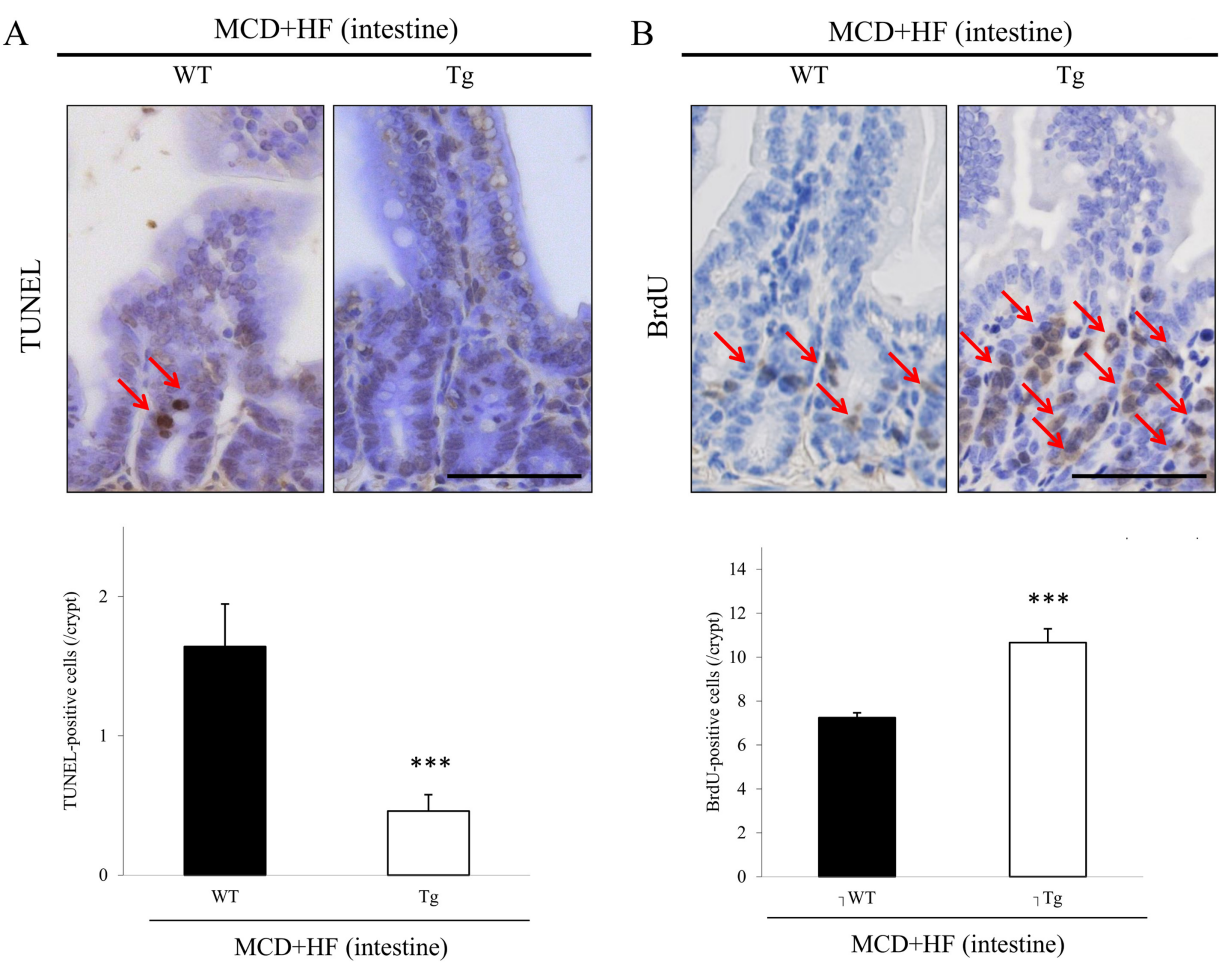
Analyses of intestinal apoptotic and proliferative activities in the MCD+HF-induced NAFLD mouse model. **A)** The number of TUNEL-positive apoptotic enterocytes in the jejunal crypts was significantly lower in model Tg mice than in model WT mice (arrows) (MCD+HF, n = 10 mice per group). Original magnification: × 400. *Bar* = 50 μm. **B)** In marked contrast, the number of BrdU-positive proliferative enterocytes in the jejunal crypts was markedly higher in model Tg mice than in model WT mice (arrows) (MCD+HF, n = 10 mice per group). Original magnification: × 400. *Bar* = 50 μm. The values are the means ± SE. ***p < 0.001.

The number of TUNEL-positive apoptotic enterocytes in the jejunal crypts was significantly lower in model Tg mice than in model WT mice (WT 1.64 ± 0.30 per crypt vs. Tg 0.46 ± 0.12 per crypt; *p* < 0.001) (Fig 6A). To label proliferating epithelial cells in the jejunal crypts two weeks after the MCD+HF diet, BrdU was subcutaneously injected into model mice 1 hr before sacrifice [29]. Contrary to the apoptotic activities (Fig 6A), the number of BrdU-positive cells was significantly larger in model Tg mice than that in model WT mice (WT 7.24 ± 0.22 per crypt vs. Tg 10.70 ± 0.63 per crypt; *p* < 0.0001) (Fig 6B).

### Analyses of Inflammatory Responses in the WT and Tg Jejunum After the Development of the MCD+HF Diet-Induced NAFLD Model

**Fig 7:**
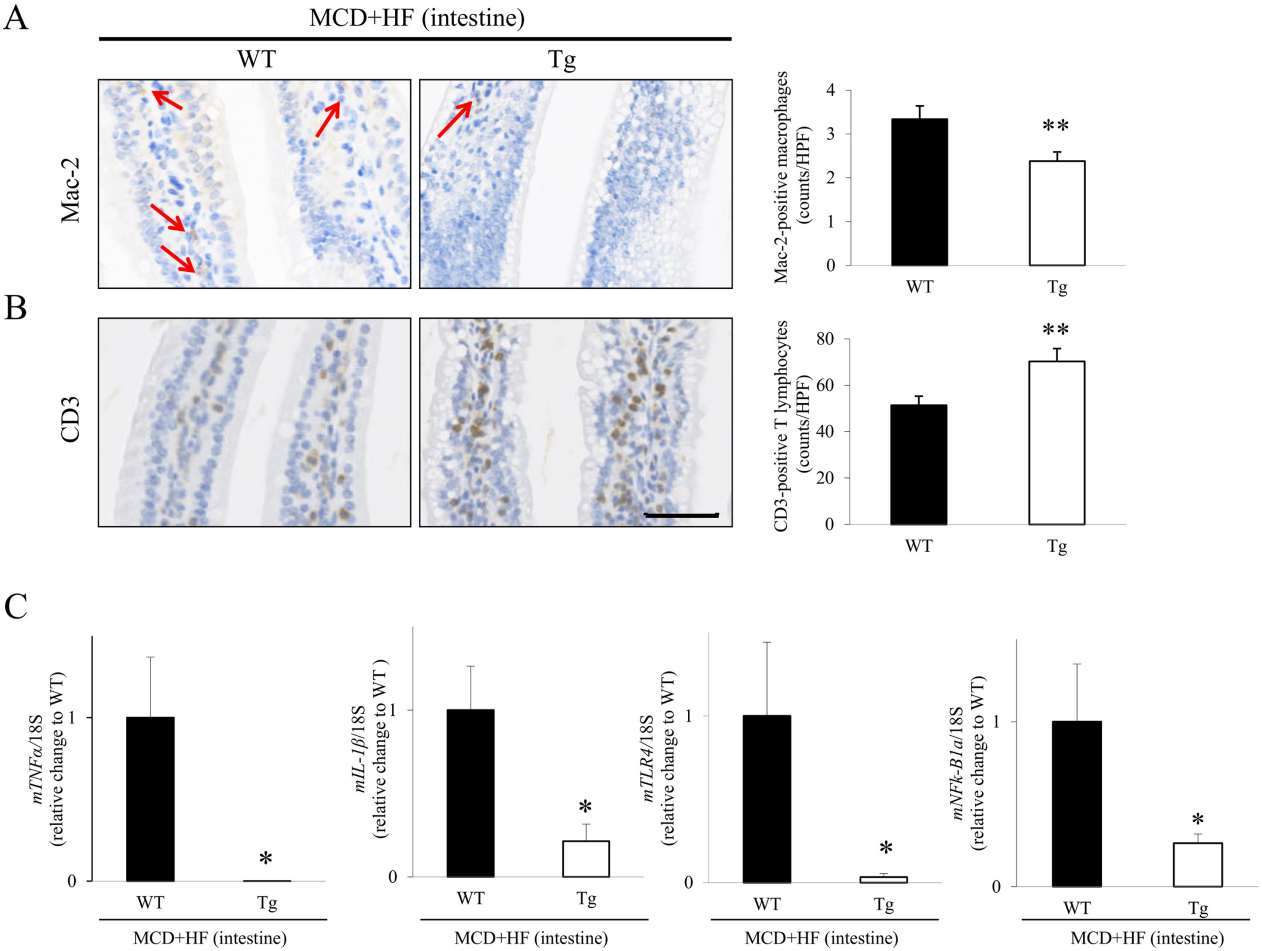
Analysis of inflammatory parameters in the small intestines of the MCD+HF-induced NAFLD mouse model. **A)** IHC showed that the number of Mac-2-positive infiltrating macrophages (arrows) in the jejunal mucosa was significantly lower in model Tg mice than in model WT mice (MCD+HF, n = 10 mice per group). **B)** Inversely, the number of CD3-positive T lymphocytes in the jejunal mucosa was significantly higher in model Tg mice than in model WT mice (MCD+HF, n = 10 mice per group). **C)** Real-time RT-PCR demonstrated that the intestinal gene expression levels of *TNF-α*, *IL-1β*, *TLR4* and *NFκ-B1a* were significantly lower in model Tg mice than those in model WT mice (MCD+HF, n = 10 mice per group). The values are the means ± SE and were normalized to the 18*S* rRNA expression (real-time RT-PCR). *p < 0.05, **p < 0.01. Original magnification: × 400. *Bar* = 50 μm.

Although a small number of inflammatory cells was observed in the jejunal mucosa in both model WT and Tg mice, IHC for Mac-2 revealed that the model Tg intestines contained a smaller number of infiltrating macrophages than the model WT intestines (WT 3.3 ± 0.3 vs. Tg 2.4 ± 0.2; *p* < 0.01) (Fig 7A). Conversely, CD3-staining showed that there were more T lymphocytes in the model Tg jejunum than in the model WT jejunum (WT 51.4 ± 3.9 vs. Tg 70.3 ± 5.5; *p* < 0.01) (Fig 7B). However, real-time RT-PCR showed that the expression levels of proinflammatory signaling factors, such as *TNF-α*, *IL-1β*, *TLR4* and *NFκ-B1a*, were significantly lower in the intestines of model Tg mice than those in model WT mice (*p* < 0.05, respectively) (Fig 7C), similar to the data in the model mouse livers (S3C Fig).

### Intestinal Expression of PDI, MTP, Cholesterol Transporters, Cholesterol Absorption Regulatory Factors and Apolipoproteins in WT and Tg Mice After the Development of the MCD+HF Diet-Induced NAFLD Model

**Fig 8:**
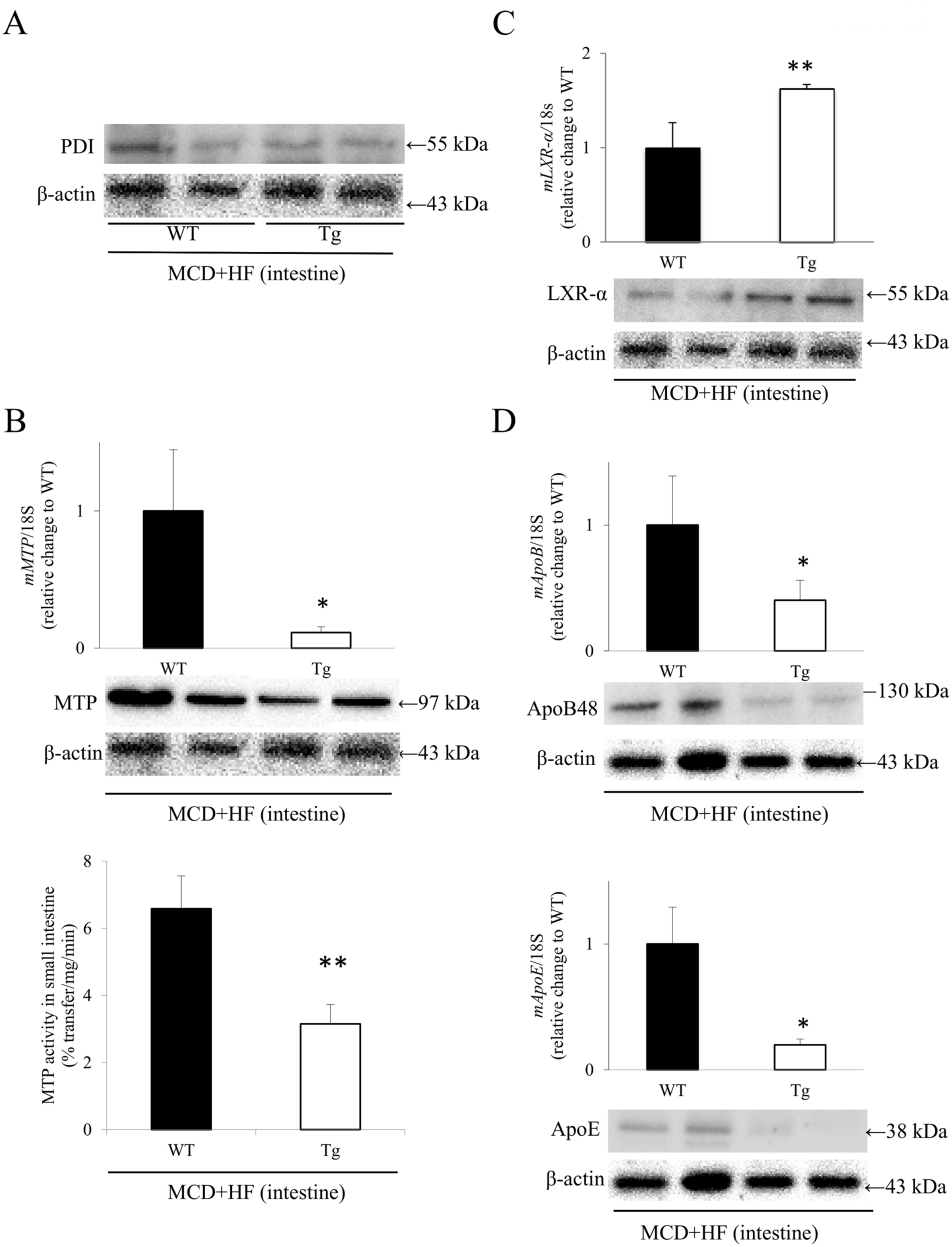
Analyses of the expression of PDI, MTP, LXR-α and apolipoproteins in the small intestines of the MCD+HF-induced NAFLD model mice. **A)** A Western blot analysis revealed that there was no significant difference in the intestinal expression levels of PDI between the model WT and Tg mice (MCD+HF, n = 10 mice per group). **B)** The real-time RT-PCR analyses (upper), Western blot analyses (middle) and assays of the lipid-transfer activity (lower) of MTP showed that the intestinal MTP expression level and its activity were significantly lower in model Tg mice than in model WT mice (MCD+HF, n = 10 mice per group). **C)** In contrast, the intestinal *LXR-α* mRNA expression levels (upper) were significantly higher in model Tg mice than those in model WT mice, which was confirmed by Western blot analysis (lower) of LXR-α (MCD+HF, n = 10 mice per group). **D)** Correspondingly, the mRNA expression levels of *ApoB* and *ApoE* (upper) were significantly lower in the small intestines of model Tg mice than those in model WT mice, which was confirmed by Western blot analysis (lower) of ApoB48 and ApoE (MCD+HF, n = 10 mice per group). The values are the means ± SE, and were normalized to the 18*S* rRNA expression (real-time RT-PCR) and β-actin expression (Western blotting). *p < 0.05, **p < 0.01.

Western blot analysis showed that the intestinal PDI expression levels were not significantly different between the two groups (Fig 8A). Contrary to the data from model mouse livers (Fig 4), real-time RT-PCR, Western blotting and MTP activity analyses demonstrated that the intestinal MTP expression levels and activity were significantly lower in model Tg mice than in model WT mice (PCR and Western blot: *p* < 0.05) (activity: WT 6.58 ± 0.98% transfer/mg/min vs. Tg 3.15 ± 0.57% transfer/mg/min; *p* < 0.01) (Fig 8B). Furthermore, the intestinal *LXR-α* mRNA expression was significantly higher in model Tg mice than that in model WT mice (*p* < 0.05; Fig 8C), as confirmed by Western blot analysis of LXR-α (Fig 8C), one of the pivotal regulatory factors in the metabolism of lipid biosynthesis [13,35]. Correspondingly, real-time RT-PCR revealed that the expression levels of *ApoB* and *ApoE* were significantly lower in the small intestines of model Tg mice than those in the model WT mouse intestines (*p* < 0.05) (Fig 8D), which was also confirmed by a Western blot analysis of ApoB48 and ApoE (Fig 8D).

**Fig 9:**
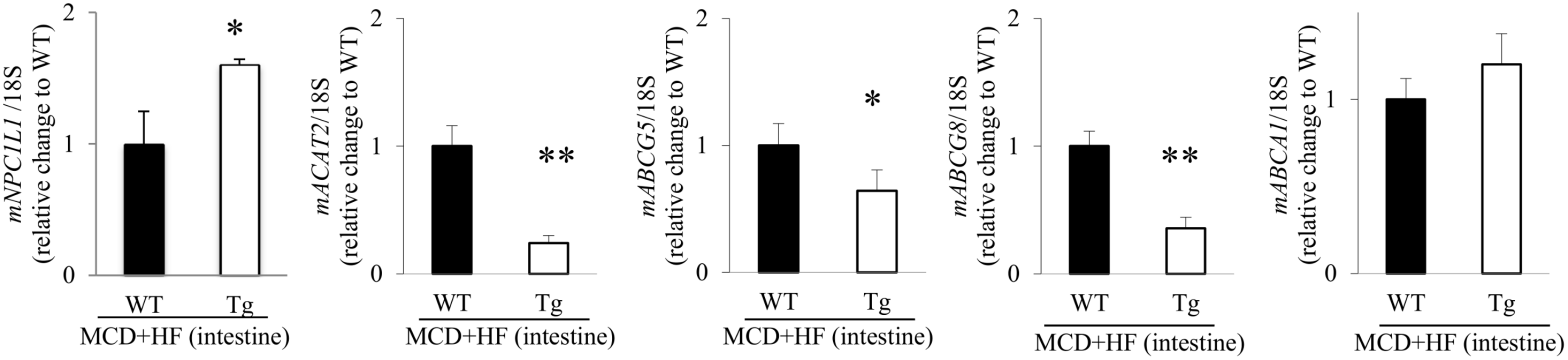
Cholesterol transporters and cholesterol absorption factor expression in the small intestines of MCD+HF-induced NAFLD model mice. The mRNA expression levels of *NPC1L1* and *ACAT2* were significantly higher in model Tg mice than in model WT mice after they had been fed the MCD+HF diet for 2 weeks, whereas the levels of *ABCG5* and *ABCG8* were significantly lower in the model Tg mice (MCD+HF, n = 10 mice per group). In contrast, the levels of *ABCA1* were not significantly different between the 2 groups of model mice. The values are the means ± SE and were normalized to the 18*S* rRNA expression (real-time RT-PCR). *p < 0.05, **p < 0.01.

In addition, real-time RT-PCR showed that the expression level of *Niemann-Pick C1 like 1 protein* (*NPC1L1*), a cholesterol-uptake transporter [13], was significantly higher in the intestines of model Tg mice than in the model WT intestines (*p* < 0.05; Fig 9). Conversely, the expression levels of *ATP-binding cassette* (*ABC*) *proteins*, *ABCG5* and *ABCG8*, cholesterol efflux transporters [13], were significantly lower in the small intestines of model Tg mice, than those in the model WT intestines (*p* < 0.05 and *p* < 0.01, respectively; Fig 9). The gene expression level of *acyl-CoA*:*cholesterol acyltransferase 2* (*ACAT2*), a cholesterol absorption-regulatory factor [13], was also significantly higher in the intestines of model Tg mice (*p* < 0.01, respectively; Fig 9). However, the level of *ABCA1*, which plays a role in controlling cholesterol absorption [13], showed no significant difference between the groups (Fig 9).

### Bacterial Counts and the Composition of Phyla/Genera in the Fecal Microbiota of WT and Tg Mice After the Development of the MCD+HF Diet-Induced NAFLD Model

The intestinal microbiota of each model mouse was analyzed in samples collected two weeks after the start of MCD+HF feeding using our unique clone library method [34]. The number of bacterial cells in each fecal sample was counted by epifluorescence staining using EtBr. The average cell counts in the model Tg fecal samples showed that there was a tendency for these samples to contain a larger number of microbiota than those of model WT mice, however, there was no significant difference (WT 1.59 ± 0.42 × 10^8^ vs. Tg 3.92 ± 1.30 × 10^8^; *p* = 0.064) (S4A Fig).

S4B Fig shows the percentage of genera containing *Bacteroides*, *Turicibacter* or *Clostridium*, and the results of a subsequent analysis of principal components, including the phyla *Bacteroidetes* and *Firmicutes*, in each fecal sample of the model mice. Although the model WT mice (Nos. 3, 4 and 5) and Tg mice (Nos. 1, 2 and 5) had only minor rates of phyla *Bacteroidetes* possession, and high rates of phyla *Firmicutes* possession major, there was no significant difference or tendencies toward difference between the two groups.

## Discussion

The overexpression of intracellular (local) and secreted (systemic) PRDX4 (i) suppressed intrahepatic/intraintestinal and circulating oxidative stressors; on one hand, (ii) suppressed apoptosis but accelerated proliferation in jejunal enterocytes; (iii) reduced intestinal inflammation; and (iv) synergistically affected intestinal function. On the other hand, it (v) reduced hepatic inflammation and ballooning; and (vi) significantly lessened the damage from NAFLD. Based on our current data in this MCD+HF diet-induced NAFLD mouse model, we could provide the first evidence that the small intestine and liver are spared to some degree by the antioxidant properties of PRDX4, and overexpression of PRDX4 beneficially affects intestinal function in the reduction of the severity of NAFLD.

Similar to our previous studies [5,6], after establishing the current dietary mouse model, we detected specific expression of hPRDX4 in the liver and small intestine of Tg mice, but no or faint expression (indicating cross-reactivity of the antibody against hPRDX4 to endogenous mPRDX4) in these tissues of WT mice. This could be supported by the fact that the cytomegalovirus (CMV) enhancer/promoter for transgenic hPRDX4 cDNA is stimulated by enhanced ROS and inflammation during the progression of NAFLD in this dietary model, via cross-talk with other promoters for many transcription factor binding sites, such as the two activator protein 1 (AP1), four NFκB and five cAMP response element binding protein (CREB) binding sites, as described previously [5,6,27]. The Tg mice revealed a significant resistance to local and systemic injury, along with maintaining markedly higher levels of tissue (i.e., liver and intestine) PRDX4 and circulating serum soluble PRDX4 protein, but inversely, had lower expression levels of endogenous mPRDXs, including mPRDX4. These suggested a complementary or negative-feedback mechanism(s) for overexpression of hPRDX4, and that the strong expression of this powerful transgene can easily overcome that of the endogenous genes in the current NAFLD model on Tg mice. Additionally, the establishment of MCD+HF feeding-induced NAFLD enhanced the oxidation by increasing the hepatic, intestinal and circulatory ROS levels, as demonstrated by the augmented DHE binding level in hepatocytes/enterocytes and serum TBARS level. Taken together, we propose that the Tg mice on the MCD+HF diet provide a good animal model for studying the protective effects of PRDX4 against the severity of NAFLD and the intestinal dysfunction by suppressing local and systemic oxidative stressors.

Corresponding to the nongenetic mouse model [6], the serum levels of lipoproteins were lower in Tg and WT MCD+HF-fed mice, while the T-cho, HDL and TG levels were significantly higher in Tg mice than in WT mice. To the best of our knowledge, very few reports have analyzed the serum lipoprotein levels as well as the small intestine features in the current animal model. The correlation between the serum lipoprotein profiles and PRDX4 overexpression still remains to be elucidated. Despite that, PRDX4 has been actually reported to play a pivotal role in the disulfide bond formation (i.e., oxidative folding) of lipoproteins in conjunction with PDI in the ER [19,36]. It is possible that PRDX4 overexpression might induce further oxidative folding and subsequent circulatory secretion of correctly folded apolipoproteins (e.g., ApoB and ApoE) from the mildly-injured liver in Tg mice. Moreover, PDI is one subunit of MTP, which has a crucial role in assembling TG droplets and apolipoproteins to form TG-rich lipoproteins in the ER of hepatocytes [13,33]. Therefore, the elevated hepatic ROS due to MCD+HF feeding in the model WT mice might cause apolipoproteins to be significantly misfolded, resulting in more pronounced NAFLD and lower serum lipoprotein profiles, compared to those in model Tg mice. Furthermore, with regard to LXR-α, which was predominantly expressed in tissues involved in lipid metabolism, such as the liver and small intestine [35,37], the present data are in agreement with the previously reported findings; activation of LXR-α plays a central role in hepatic lipogenesis, causing hepatic steatosis and the development of intrahepatic inflammation [38]. However, these intriguing issues related to lipid metabolism should be examined in more detail after accumulating further experimental data.

The small intestine plays a critical role in the efficient absorption and metabolic processing of nutrients, and the enterocytes are responsible for sensing the luminal contents modulated by the diet, especially a HF diet [12,13]. The morphological, biochemical and molecular analyses revealed that intestinal lipid deposits in the surface enterocytes, as well as the TG, FFA and CE accumulation, were significantly increased in the model Tg mice. These findings were accompanied by the presence of fewer apoptotic, but more proliferating, epithelial cells, particularly in the crypts. As apoptosis can trigger aberrant regenerative and proliferative responses in the multipotent stem cells located in the intestinal crypts, this may result in uncontrolled cell-renewal processes, including migration or differentiation, and the subsequent shortening of the jejunal villi height and the total length [39], as shown in the model WT (but not Tg) mice. In fact, functional mature enterocytes in the villi of adult mice are known to be renewed every three to five days from those crypt stem cells, and thus, the dysregulated renewal process would lead to significantly decreased intestinal fat absorption capacity at the mucosal surface, where nutrient absorption mainly takes place [39]. Furthermore, we found fewer infiltrating jejunal macrophages and lower expression of various inflammatory cytokines in the intestines of model Tg mice. It is possible that more jejunal CD3-positive cells in the model Tg mice might be regulatory T lymphocytes, which have a central function in controlling the strictly regulated intestinal immune system and intestinal commensal microbiota [40]. Inflammation and apoptotic activities in the intestinal epithelium are responsible for disrupting the mucosal integrity and barrier function to enterobacterial invasion, which is associated with a significant increase in the expression of intestinal and hepatic *TLR4*, *NFκ-B* and/or *TNF-α* genes [14,15,41]. Indeed, human NASH patients present a higher prevalence of small intestine bacterial overgrowth and a lower proportion of the phyla *Bacteroidetes*, with increased gut permeability [15]. Since these findings appear to be in disagreement with the present data from bacterial counts and the composition of the phyla/genus in the mouse fecal microbiota, further experiments are necessary to address these complicated issues, including the species difference between humans and mice.

Regardless, according to our recent findings in an animal model of inflammatory bowel disease [42], macrophages should play a key role in the intestinal inflammatory response, especially via upregulated expression of TNF-α, but the apoptotic activity of the intestinal epithelium can also have a close relationship with aberrant intestinal inflammation, and *vice versa*. Furthermore, increased DHE associated fluorescence in the jejunum, and the circulating TBARS levels were significantly reduced in the model Tg mice in the present study. Taken together, these data suggest that the overexpression of hPRDX4 may protect against crypt apoptosis, chronic inflammation and subsequent dysfunction of the small intestine, in the suppression of NAFLD progression, at least partly by preventing apoptotic and inflammatory cells-derived ROS generation and scavenging extracellular ROS [5,6,25]. We have actually found that oxidative stress is closely associated with apoptosis, as previously shown in other organs, such as the pancreas, arterial wall and liver [5,6,25]. These collective findings from the Tg mice manifest as potentially beneficial effects of the intestinal function, rather than detrimental mechanisms that promote metabolic disorders [33].

In marked contrast to the data from model mouse livers, the intestinal MTP and ApoB/E expression levels and the MTP activity were significantly lower, but the intestinal LXR-α expression was higher, in the model Tg mice than in the WT mice. LXR-α is one of the master regulatory factors in lipid catabolism, driving the elimination of cholesterol from the periphery to the lumen of the intestine [13,37]. Our results are in agreement with recently published data from another group using zebrafish larvae showing that the overexpression of intestinal LXR-α delays the appearance of ingested lipids to the vasculature, which is associated with the storage of absorbed lipids in enterocytes [43]. Although the absorption of lipids from the intestinal lumen into the enterocytes and their subsequent secretion into the circulation is a complex process, membrane transporters regulate the lipid uptake on the apical surface of the brush border of enterocytes [13]. Corresponding to the pivotal roles for NPC1L1 in cholesterol uptake and for the coordinated activities of ABCG5 and ABCG8 in cholesterol efflux, the intestinal expression profiles of these cholesterol transporters and cholesterol absorption-regulatory factors in the model Tg mice might support the histopathological and biochemical findings. Furthermore, since the ApoB and MTP activity in the ER are also known to play a crucial role in the efficient assembly and secretion of lipoproteins into chylomicrons [13,33], the present data could be in line with the beneficial effects of intestinal function in the Tg mice during the development of the MCD+HF diet-induced NAFLD model. However, contrary to our data, increased expression of LXR-α in the intestine leads to reduction of intestinal cholesterol absorption via lower intestinal NPC1L1 expression and higher intestinal ABCG5 and ABCG8 expression [44]. Nevertheless, further studies are needed to clarify those issues, since most of our present data are obtained from gene and protein expressions.

In fact, the present study using a dietary mouse model would contain several limitations in its interpretation: (i) a snapshot of one particular time, (ii) no detailed measurements of lipid flux (e.g., TG and/or cholesterol absorption, and fecal lipid and bile acid contents), and thus, finally, (iii) none of thorough experiments regarding lipid metabolism. On the other hand, in our future studies, we should compare expression patterns of the different transporters in Tg mice fed normal chow diet, with those in Tg mice fed a HF diet with or without the MCD diet. This would add tremendously to the understanding of the physiological change in the intestinal function brought on by PRDX4 overexpression, irrespective of the MCD diet.

In conclusion, hPRDX4 Tg mice showed the beneficial effects of the antioxidant properties on intestinal function in the suppression of MCD+HF-induced NAFLD. Our accumulated data indicate that hPRDX4 overexpression plays critical roles in (i) suppressing local (intrahepatic and intraintestinal) and systemic (circulating) oxidative stressors, (ii) affecting possibly beneficial intestinal function by suppressing crypt enterocyte apoptosis and inflammation, and by attenuating the shortening of the total length and villi height, and (iii) reducing the severity of NAFLD by suppressing inflammation and ballooning. Both small intestine and liver would be spared to some degree by the antioxidant properties of PRDX4. It is also noteworthy that activators of PRDX4 could offer therapeutic potential for ameliorating the severity of NAFLD and intestinal dysfunction by suppressing oxidative damage.

## Supporting Information

S1 Fig Analysis of the expression of PRDXs mRNA in the model mice. **A)** Real-time RT-PCR confirmed that the hPRDX4 expression was markedly higher in the livers and intestines from MCD+HF model Tg mice (n = 10 mice per group). **B)** The hepatic and intestinal expression levels of endogenous mPRDXs were significantly lower in model Tg mice than in model WT mice. However, the hepatic expression level of mPRDX4 was not significantly different between model WT and Tg mice (MCD+HF, n = 10 mice per group). The values are the means ± SE and were normalized to the 18*S* rRNA expression (real-time RT-PCR). *p < 0.05, **p < 0.01, ***p < 0.001.
(TIF)

S2 Fig Analysis of hPRDX4 expression and oxidative stress markers in the NAFLD livers of model mice. IF demonstrated that the numbers of increased DHE associated fluorescence (*red-stained*) within hepatocytes were significantly higher in the model livers from WT mice than in those from Tg mice (MCD+HF, n = 10 mice per group). Correspondingly, IHC revealed that hPRDX4 was detected in a number of hepatocytes (arrows) throughout the livers of model Tg mice, but not those from model WT mice. Original magnification: × 400. *Bar* = 50 μm. The values are the means ± SE. **p < 0.01.
(TIF)

S3 Fig Analysis of inflammatory parameters in the NAFLD livers of the MCD+HF model. **A)** IHC showed that the number of Mac-2-positive macrophages (Kupffer cells) in the liver was significantly different between model WT and Tg mice (MCD+HF, n = 10 mice per group). **B)** Similarly, the number of CD3-positive infiltrating T lymphocytes in the liver was significantly lower in model Tg mice than in model WT mice (MCD+HF, n = 10 mice per group). **C)** Real-time RT-PCR showed that the hepatic gene expression levels of *TNF-α*, *IL-1β*, *TLR4* and *NFκ-B1a* were significantly lower in model Tg mice than those in model WT mice (MCD+HF, n = 10 mice per group). The values are the means ± SE and were normalized to the 18*S* rRNA expression (real-time RT-PCR). *p < 0.05, **p < 0.01. Original magnification: × 400. *Bar* = 50 μm.
(TIF)

S4 Fig Bacterial counts and the phylum/genus composition in the mouse fecal microbiota after the development of the MCD+HF diet-induced NAFLD model. **A)** The number of bacterial cells in each fecal sample from model mice (MCD+HF, n = 5 mice per group) was counted by epifluorescence staining using EtBr. The average cell counts in the model Tg fecal sample showed a tendency to contain a larger number of microbiota than those of model WT mice, however, no significance was noted for the difference between groups. **B)** The percentage of genera containing *Bacteroides*, *Turicibacter* or *Clostridium* (right), and the subsequent analysis of principal components including the phyla *Bacteroidetes* and *Firmicutes* (left), in each fecal sample from model mice are shown. Although the model WT mice (Nos. 3, 4 and 5) and Tg mice (No. 1, 2 and 5) had low rates of phyla *Bacteroidetes* possession and high rates of phyla *Firmicutes* possession, there was neither significant difference nor tendencies toward significant difference between the 2 groups.
(TIF)

S1 Table The RT-PCR and real-time RT-PCR primers used in the study. (TIF)

## Abbreviations

ABC: ATP-binding cassette
ACAT: acyl-CoA:cholesterol acyltransferase
Apo: apolipoprotein
ALT: alanine aminotransferase
AST: aspartate aminotransferase
BrdU: 5’-Bromo-2’-deoxyuridine
BW: body weight
CE: cholesteryl ester
DHE: dihydroethidium
ELISA: enzyme-linked immunosorbent assay
ER: endoplasmic reticulum
EtBr: ethidium bromide
FFA: free fatty acid
H&E: hematoxylin and eosin
HF: high fat diet
hPRDX4: human peroxiredoxin 4
IL: interleukin
LDL: low-density lipoprotein
LXR: liver X receptor
MCD+HF: methionine- and choline-deficient high fat diet
MDA: malondialdehyde
mPRDX4: mouse peroxiredoxin 4
MTP: microsomal triglyceride-transfer protein
NAFLD: nonalcoholic fatty liver disease
NASH: nonalcoholic steatohepatitis
NFκ-B: nuclear factor κ-B
NPC1L1: Niemann-Pick C1 like 1 protein
PDI: protein disulfide isomerase
PRDX: peroxiredoxin
ROS: reactive oxygen species
RT-PCR: reverse transcriptase-polymerase chain reaction
Tg: hPRDX4 transgenic
TG: triglyceride
TLR: toll-like receptor
TNF: tumor necrosis factor
TUNEL: terminal deoxynucleotidyl transferase end-labeling
WT: wild-type
