# Supplementary figures and images for: Overexpression of Peroxiredoxin 4 Affects Intestinal Function in a Dietary Mouse Model of Nonalcoholic Fatty Liver Disease
Source: PLoS One. 2016 Apr 1;11(4):e0152549. doi: 10.1371/journal.pone.0152549 (PMC4818088; doi:10.1371/journal.pone.0152549)

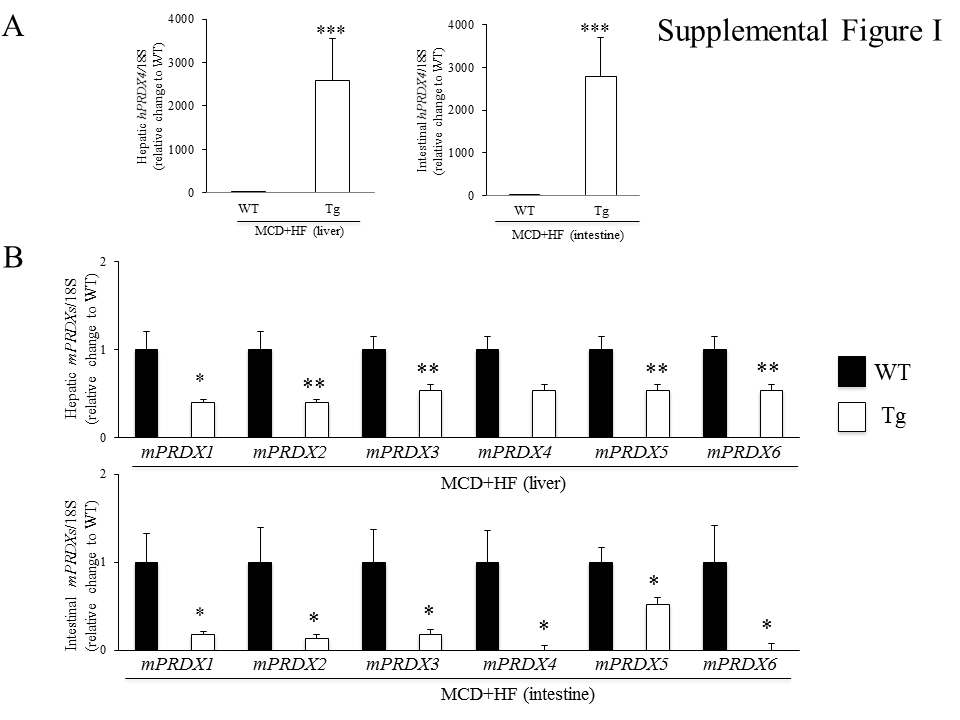

Supplement: S1 Fig — A) Real-time RT-PCR confirmed that the hPRDX4 expression was markedly higher in the livers and intestines from MCD+HF model Tg mice (n = 10 mice per group). B) The hepatic and intestinal expression levels of endogenous mPRDXs were significantly lower in model Tg mice than in model WT mice. However, the hepatic expression level of mPRDX4 was not significantly different between model WT and Tg mice (MCD+HF, n = 10 mice per group). The values are the means ± SE and were normalized to the 18S rRNA expression (real-time RT-PCR). *p < 0.05, **p < 0.01, ***p < 0.001. (TIF) [file pone.0152549.s001.tif]

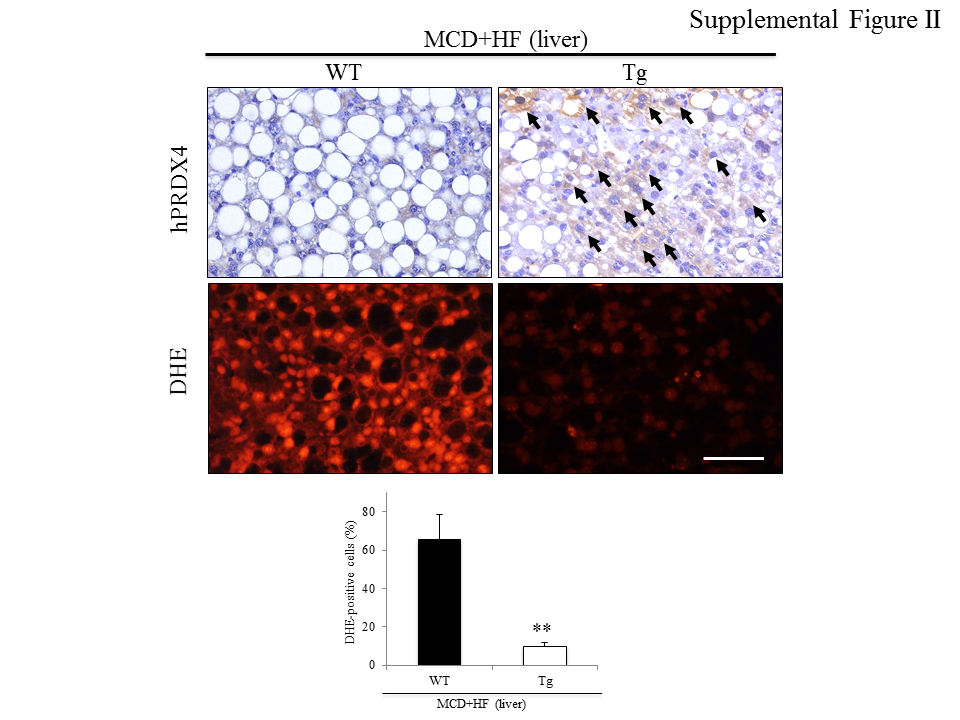

Supplement: S2 Fig — IF demonstrated that the numbers of increased DHE associated fluorescence (red-stained) within hepatocytes were significantly higher in the model livers from WT mice than in those from Tg mice (MCD+HF, n = 10 mice per group). Correspondingly, IHC revealed that hPRDX4 was detected in a number of hepatocytes (arrows) throughout the livers of model Tg mice, but not those from model WT mice. Original magnification: × 400. Bar = 50 μm. The values are the means ± SE. **p < 0.01. (TIF) [file pone.0152549.s002.tif]

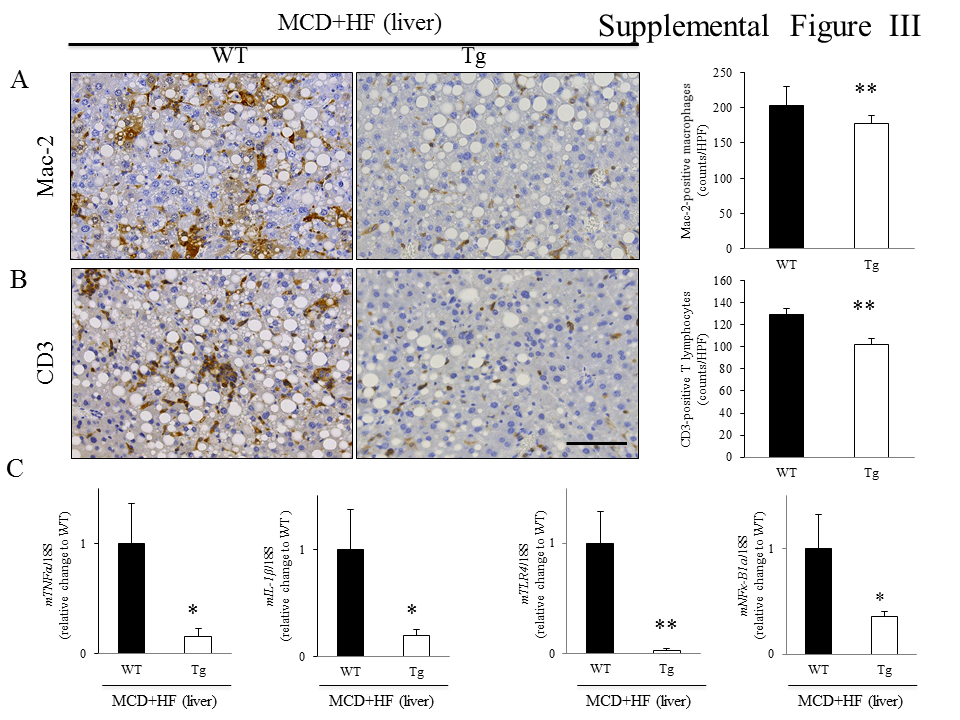

Supplement: S3 Fig — A) IHC showed that the number of Mac-2-positive macrophages (Kupffer cells) in the liver was significantly different between model WT and Tg mice (MCD+HF, n = 10 mice per group). B) Similarly, the number of CD3-positive infiltrating T lymphocytes in the liver was significantly lower in model Tg mice than in model WT mice (MCD+HF, n = 10 mice per group). C) Real-time RT-PCR showed that the hepatic gene expression levels of TNF-α, IL-1β, TLR4 and NFκ-B1a were significantly lower in model Tg mice than those in model WT mice (MCD+HF, n = 10 mice per group). The values are the means ± SE and were normalized to the 18S rRNA expression (real-time RT-PCR). *p < 0.05, **p < 0.01. Original magnification: × 400. Bar = 50 μm. (TIF) [file pone.0152549.s003.tif]

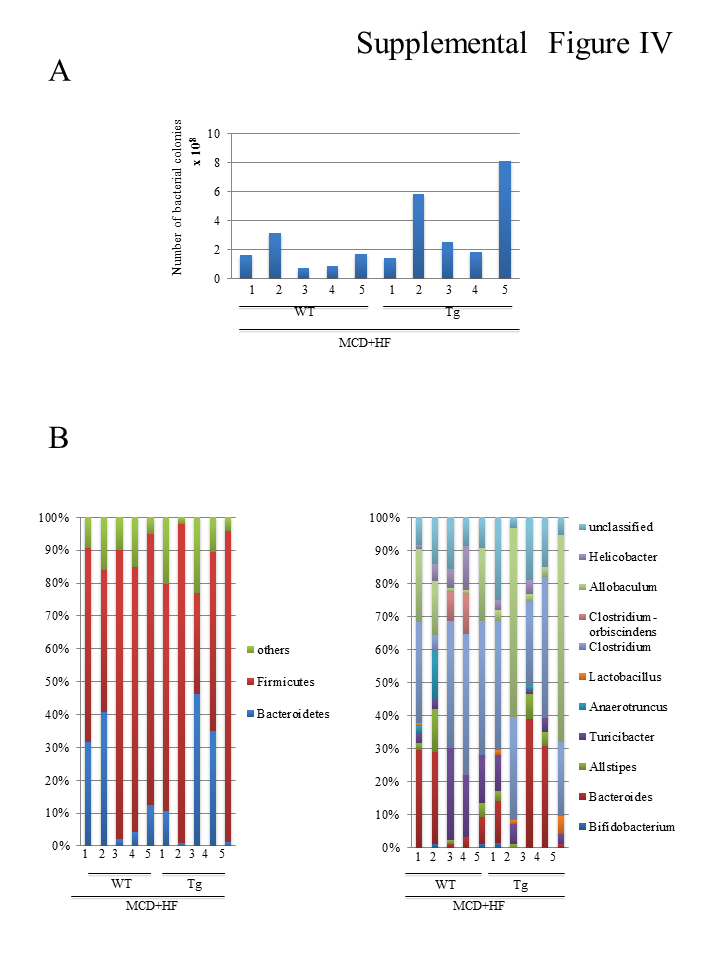

Supplement: S4 Fig — A) The number of bacterial cells in each fecal sample from model mice (MCD+HF, n = 5 mice per group) was counted by epifluorescence staining using EtBr. The average cell counts in the model Tg fecal sample showed a tendency to contain a larger number of microbiota than those of model WT mice, however, no significance was noted for the difference between groups. B) The percentage of genera containing Bacteroides, Turicibacter or Clostridium (right), and the subsequent analysis of principal components including the phyla Bacteroidetes and Firmicutes (left), in each fecal sample from model mice are shown. Although the model WT mice (Nos. 3, 4 and 5) and Tg mice (No. 1, 2 and 5) had low rates of phyla Bacteroidetes possession and high rates of phyla Firmicutes possession, there was neither significant difference nor tendencies toward significant difference between the 2 groups. (TIF) [file pone.0152549.s004.tif]

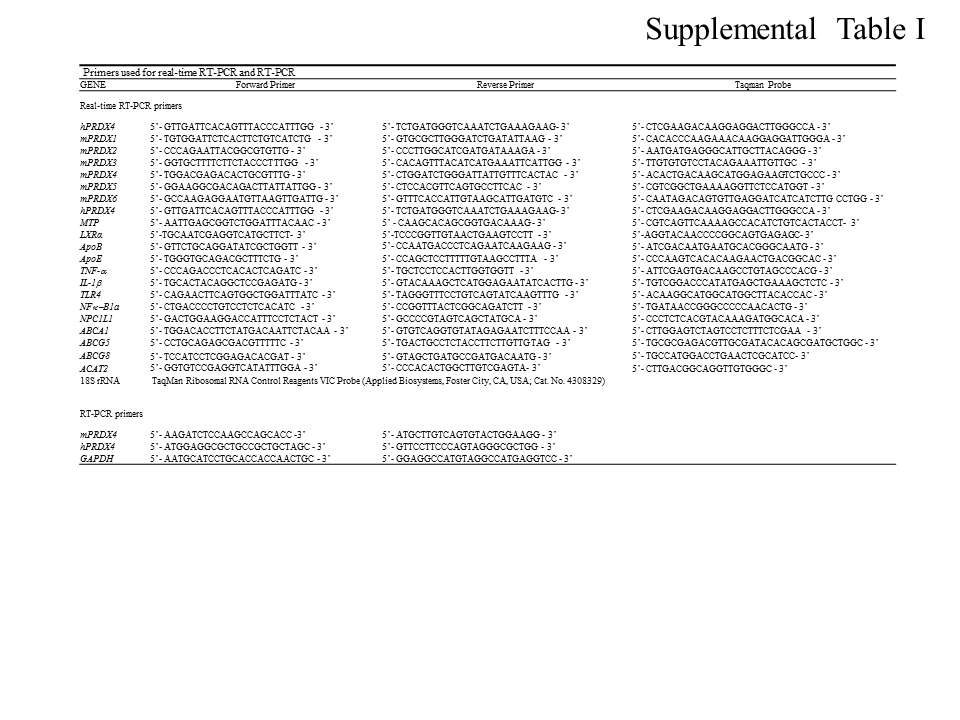

Supplement: S1 Table — (TIF) [file pone.0152549.s005.tif]
